# Occupation and mesothelioma in Sweden: updated incidence in men and women in the 27 years after the asbestos ban

**DOI:** 10.4178/epih.e2016039

**Published:** 2016-09-20

**Authors:** Nils Plato, Jan Ivar Martinsen, Pär Sparén, Gunnar Hillerdal, Elisabete Weiderpass

**Affiliations:** 1 Institute of Environmental Medicine, Karolinska Institutet, Stockholm, Sweden; 2 Department of Research, Cancer Registry of Norway, Institute of Population-Based Cancer Research, Oslo, Norway; 3 Department of Medical Epidemiology and Biostatistics, Karolinska Institutet, Stockholm, Sweden; 4 Department of Medicine, Karolinska Institutet, Stockholm, Sweden; 5 Department of Community Medicine, Faculty of Health Science, University of Tromsø, The Arctic University of Norway, Tromsø, Norway; 6 Genetic Epidemiology Group, Folkhälsan Research Center, Helsinki, Finland

**Keywords:** Epidemiology, Asbestos, Job-exposure matrix, Carcinogenic substances

## Abstract

**OBJECTIVES:**

We updated the Swedish component of the Nordic Occupational Cancer (NOCCA) Study through 2009 in order to investigate the incidence of mesothelioma of the peritoneum and pleura in both genders, and explored occupational exposures that may be associated with mesothelioma.

**METHODS:**

The Swedish component of the NOCCA Study includes 6.78 million individuals. Data from this cohort were linked to the population-based Swedish Cancer Registry and Swedish Total Population Registry for three periods between 1961 and 2009, and then further linked to the Swedish NOCCA job-exposure matrix, which includes 25 carcinogenic substances and the corresponding exposure levels for 280 occupations. Multivariate analysis was used to calculate standardized incidence ratios (SIRs) for mesothelioma of the peritoneum and pleura by gender, occupational category, carcinogenic substance, and for multiple occupational exposures simultaneously.

**RESULTS:**

A total of 3,716 incident mesotheliomas were recorded (21.1% in women). We found a significantly increased risk of mesothelioma in 24 occupations, as well as clear differences between the genders. Among men, increased risks of mesothelioma of the pleura were observed in male-dominated occupations, with the greatest elevation of risk among plumbers (SIR, 4.99; 95% confidence interval, 4.20 to 5.90). Among women, increased risks were observed in sewing workers, canning workers, packers, cleaners, and postal workers. In multivariate analysis controlling for multiple occupational exposures, significant associations were only observed between asbestos exposure and mesothelioma.

**CONCLUSIONS:**

Asbestos exposure was associated with mesothelioma incidence in our study. The asbestos ban of 1982 has yet to show any clear effect on the occurrence of mesothelioma in this cohort. Among women, the occupations of canning workers and cleaners showed increased risks of mesothelioma of the pleura without evidence of asbestos exposure.

## INTRODUCTION

Mesothelioma is a very aggressive malignancy that occurs in the peritoneum and pleura, and more rarely in the pericardium. It is most often linked to occupational asbestos exposure and was classified as an occupational disease in the 1950s [1]. Mesothelioma has a poor prognosis, with most patients dying within one year of diagnosis [2], and a long latency period of up to 40 years [3]. The incidence of mesothelioma varies; its incidence in developed countries such as Belgium, Britain, and Australia exceeds 30 cases per one million inhabitants [4], and in Sweden approximately 12 cases are diagnosed per one million inhabitants (approximately 120 cases per year) [5].

Mesothelioma development is most strongly associated with exposure to asbestos types from the amphibole family, of which crocidolite was the most prevalent type used in Sweden. Therefore, occupations in which crocidolite was used or manipulated constituted high-risk occupations. In some industries, such as asbestos-cement production, asbestos types from both the serpentine and amphibole families were used [6].

Asbestos was banned in Sweden in 1982 [7], and strict precautions and security requirements have been introduced for occupations that include the handling of or exposure to asbestos, such as asbestos removal. A sharp decline in the annual incidence rate of mesothelioma was observed in 2008 [8], but in the last two years its incidence has started to increase again. One of the reasons for this recent increase may be the long latency period of mesothelioma; however, since asbestos has been absent from the Swedish labor market for 35 years, one would expect the number of mesothelioma cases to be on the decline. Instead, at present more people in Sweden die from mesothelioma than from accidents in the workplace [9].

Only a limited number of occupations have been reported to confer a significant excess risk of mesothelioma [10], and no connection between mesothelioma and any occupational exposure besides asbestos, such as air pollution [11], has been scientifically proven. In the UK and Finland, it has been estimated that 97% [12] and 90% [13] of mesothelioma cases, respectively, are related to asbestos exposure, and most of these are caused by occupational exposure.

The most recent report on the Nordic Occupational Cancer (NOCCA) cohort covered the period from 1961 to 2005 and is the largest study of occupational cancer in the Nordic countries published so far. It included 2.8 million diagnosed cases of cancer in the five Nordic countries (Denmark, Finland, Iceland, Norway, and Sweden) and reported mesothelioma cases in occupations where asbestos exposure is not usually considered to be present [14]. A total of 40.6% of the mesothelioma cases in the NOCCA cohort were from Sweden (2,521 men and 548 women), and a statistically significant excess risk of mesothelioma among men in Sweden was found in 12 of the 53 occupational categories considered [14]. However, the proportion of women diagnosed with mesothelioma was also quite high.

Asbestos exposure alone probably cannot explain the relatively high proportion of women with mesothelioma in Sweden, since disease onset was found to occur at approximately 50 years of age, which is much earlier than the onset for men [5]. Moreover, asbestos exposure during the period covered by the NOCCA Study was most common in male-dominated occupations (e.g., insulators, ship builders, car mechanics, asbestos cement manufacturing, construction workers, etc.) [15]. However, since 17.8% of the mesothelioma cases in the latest Swedish NOCCA Study occurred in women [14], it is important to identify alternative direct or indirect causal factors.

Therefore, we updated the data in the Swedish component of the NOCCA Study through 2009 in order to investigate the incidence of mesothelioma of the peritoneum and pleura in both genders, and to explore occupational exposures other than asbestos that may be associated with mesothelioma. We also investigated whether mesothelioma incidence during the period from 1961 to 2009 differed among occupations and whether it was possible to see any effect of the Swedish asbestos ban of 1982.

## MATERIALS AND METHODS

### The Swedish Nordic Occupational Cancer Study

The NOCCA Study was started to investigate occupational exposures and their associations with cancer incidence. The Swedish component of the NOCCA Study consists of individuals who participated in one of the following Swedish population censuses: 1960, 1970, 1980, or 1990. The 1960, 1970, and 1990 censuses took place on November 1, while the 1980 census took place on September 15. The Swedish NOCCA Study included 6.78 million individuals aged 30 to 64 years who were still alive and living in Sweden on January 1 of the year following the census. Each individual’s personal identification number, name, address, marital status, education, economic activity, occupation, and industry were recorded, and the occupations were then coded, as described previously by Pukkala et al. [14], according to a Swedish adaptation of the Nordic Occupational Classification [16], which is a Nordic adaptation of the International Standard Classification of Occupations from 1958 [17].

### Update of cancer incidence through 2009

Cancer incidence was determined through record linkage between the Swedish NOCCA database and the population-based Swedish Cancer Registry. The present report updated information on cancer incidence in subjects included in the Swedish NOCCA Study through 2009, thus covering the period from 1961 to 2009. Follow-up was done over three time periods: 1961-1974, 1975-1989, and 1990-2009. Mesothelioma cases were classified into mesothelioma of the peritoneum (International Classification of Diseases, 7th revision [ICD-7] code 158) and of the pleura (ICD-7 code 162.2). Histological results were coded with three digits by the Swedish Cancer Registry (code 776), according to the official codes of the World Health Organization [18].

Study entry was set at January 1 of the year after the first available census in which an individual participated. Person-years were accumulated until the date of emigration, death, or the end of the study period (December 31, 2009), whichever came first. Dates of death and emigration were retrieved from the Swedish Total Population Registry.

### Standardized incidence ratio

The observed number of mesothelioma cases and person-years were stratified into 5-year age categories (30-34, 35-39, … , ≥85 years) and 5-year calendar periods by gender and occupational category. Standardized incidence ratios (SIRs) were calculated using cancer incidence rates for the entire Swedish population by gender, age, and calendar time as reference rates. The exact 95% confidence interval (CI) was defined assuming a Poisson distribution of the observed number of cases.

### The Nordic Occupational Cancer job-exposure matrix

The NOCCA job-exposure matrix (NOCCA-JEM) contains combined information from all the Nordic countries. It includes 25 carcinogenic substances with exposure levels for 280 occupations based on exposure data from national exposure databases and expert assessments available from 1945 to 1994 [19]. The NOCCA-JEM is quantitative; therefore, in principle it is possible to estimate the cumulative exposure (dose) of the 25 included carcinogenic substances for the entire NOCCA cohort.

The Finnish JEM (FINJEM) [20] was created by the Finnish Institute of Occupational Health in Helsinki, Finland. This is a generic quantitative JEM from 1945 onwards for 84 different outcomes, with exposures for almost 300 different occupations at 3-digit levels. This JEM is now used in many different countries. The FINJEM was used to construct national NOCCA JEMs for Denmark, Finland, Iceland, Norway, and Sweden. The exposure codes, definitions, and units of the carcinogenic substances included in the Swedish NOCCA-JEM can be found in Appendix 1. The Swedish NOCCA-JEM uses the 3-digit occupation codes used in the Swedish adaptation of the Nordic Occupational Classification. The data of the Swedish NOCCA Study were linked to the Swedish NOCCA-JEM to determine the exposures that occurred in the different occupations. Exposure information that went beyond the confines of the NOCCA-JEMs was obtained by an experienced occupational hygienist. Of the 280 occupational codes in the Swedish NOCCA Study, 96 were rated for occupational exposure to carcinogenic substances in the Swedish NOCCA-JEM.

Finally, mesothelioma incidence was linked with the Swedish NOCCA-JEM [19] and the observed number of cases was recorded by gender and by exposure to the carcinogenic substances included in the Swedish NOCCA-JEM.

The Swedish NOCCA Study received ethics committee/institutional review board approval from the Forskningsetikkommitten at the Karolinska Institutet (Dnr 03-466).

## RESULTS

A total of 3,716 cases of mesothelioma were diagnosed in the cohort of the Swedish NOCCA Study from 1961 to 2009, 785 (21.1%) of which occurred in women. The vast majority of cases (89.7%) had mesothelioma of the pleura, while a small proportion was diagnosed with mesothelioma of the peritoneum (10.3%). However, only 577 of the 3,334 cases of mesothelioma of the pleura occurred in women (17.3%), as opposed to 208 of the 382 (54.5%) cases of mesothelioma of the peritoneum. We found a significant excess risk of incident mesothelioma of both the peritoneum and pleura in 24 of the 280 occupations considered. Differences in risk between the genders were also found. Five occupations showed an excess risk in women, but no excess risk in men (Tables 1 and 2).

For men, the linkage of cancer incidence with the Swedish NOCCA-JEM resulted in the identification of 17 occupations with chemical exposure that conferred a significant excess risk of mesothelioma of the pleura, four of which also showed an excess risk of mesothelioma of the peritoneum: plumbers and pipe fitters (pleura: SIR, 4.99; 95% CI, 4.20 to 5.90; peritoneum: SIR, 3.56; 95% CI, 1.31 to 7.76), painters (pleura: SIR, 1.72; 95% CI, 1.35 to 2.16; peritoneum: SIR, 2.70; 95% CI, 1.08 to 5.56), bricklayers (pleura: SIR, 1.60; 95% CI, 1.07 to 2.30; peritoneum: SIR, 5.42; 95% CI, 1.99 to 11.80), and insulators (pleura: SIR, 10.9; 95% CI, 6.81 to 16.50; peritoneum: SIR, 64.70; 95% CI, 28.00 to 128.00) (Table 1, Appendix 2). Twelve of the 17 occupations with excess risk are rated for asbestos exposure in the Swedish NOCCA-JEM. However, most of these occupations are also rated for other exposures in the Swedish NOCCA-JEM. Moreover, the Swedish NOCCA-JEM indicates that the observed excess risk for mesothelioma of the pleura among mechanical engineers and technicians (SIR, 1.67; 95% CI, 1.42 to 1.95); toolmakers, machine-tool setters, and operators (SIR, 1.48; 95% CI, 1.22 to 1.77); chemical process workers (SIR, 2.15; 95% CI, 1.20 to 3.54); paper pulp workers (SIR, 2.10; 95% CI, 1.22 to 3.36); and stationary engine and related equipment operators (SIR, 1.77; 95% CI, 1.05 to 2.79) is not sufficiently high to classify them as occupations that include asbestos exposure (Table 1). An elevated risk of mesothelioma of the pleura was also observed in two occupations that are not rated for chemical exposure in the Swedish NOCCA-JEM: ships’ engineers (SIR, 6.06; 95% CI, 3.65 to 9.46), and divers and pipe layers (SIR, 4.20; 95% CI, 1.14 to 10.7) (Table 1, Appendix 3). The highest risk of mesothelioma of the pleura was observed for insulators (SIR=10.9), and the largest number of cases was observed among machinery fitters and machinery assemblers (n=180).

When women with mesothelioma were linked to the Swedish NOCCA-JEM, we observed an excess risk of mesothelioma of the pleura for one occupation rated for chemical exposure: sewing work (SIR, 2.14; 95% CI, 1.27 to 3.39) (Table 2, Appendix 4). We observed significant excess risks of mesothelioma of the pleura in two occupations not rated for chemical exposure in the Swedish NOCCA-JEM: canning workers (SIR, 4.90; 95% CI, 1.33 to 12.50) and cleaners (SIR, 1.59; 95% CI, 1.05 to 2.31). In total, 208 women had mesothelioma of the peritoneum, of whom 104 were economically inactive. The other 104 women were distributed among 280 occupations, of which only two had a significant excess risk: sorting clerks and postal workers (SIR, 9.27; 95% CI, 1.12 to 33.50) and packers (SIR, 4.49; 95% CI, 1.22 to 11.50) (Table 2, Appendix 5).

We examined the trends over the three follow-up periods: 1961-1974, 1975-1989, and 1990-2009. The significant excess risk of mesothelioma of the peritoneum was constant over time for plumbers and pipefitters as well as for electrical fitters and wiremen. For sheet metal workers and electrical fitters and wiremen, we observed an increased risk during all three follow-up periods, while we observed a decreased risk for stationary engine and related equipment operators, mechanical engineers and technicians, machinery fitters and machinery assemblers, welding and flame cutters, and ships’ engineers among men, and sewing work among women (Table 3). For mesothelioma of the peritoneum, we observed a decreased risk for insulators but no consistent trends for any other occupation (Table 4).

## DISCUSSION

Mesothelioma is closely related to asbestos exposure [1,21]. Despite the ban on asbestos put in place in Sweden in 1982 [7], the incidence rate of mesothelioma increased in subsequent decades, flattened out in 2000, and started to decrease after 2005 [8]. A total of 3,716 individuals in the Swedish NOCCA Study were diagnosed with mesothelioma from 1961 to 2009. The predominant type of mesothelioma in both genders was mesothelioma of the pleura, which was also much more common in men than in women. The proportion of women with mesothelioma of the pleura was 17.8%, but when mesothelioma of the peritoneum was included, the proportion of women with mesothelioma increased to 21.1%. Only 0.7% of all cases of mesothelioma are pericardial mesothelioma [22].

Men in traditionally male-dominated occupations, such as sheet metal workers, plumbers, welders, and others, had a significantly increased risk, while we did not observe any cases of mesothelioma among women in those occupations. This is probably due to the fact that there are few women in these occupations, and that job activities and exposure patterns can differ between genders. In occupations dominated by women, significantly increased risks were found among packers, sewing workers, cleaners, canning workers, and sorting clerks and postal workers. Sewing work showed the highest increased risk of mesothelioma of the pleura in the first period (1961-1974) (SIR, 5.65; 95% CI, 1.54 to 14.50), the second period (1975-1989) (SIR, 3.07; 95% CI, 1.40 to 5.83), and the full period (1961-2009) (SIR, 2.14; 95% CI, 1.27 to 3.39). Sewing workers are exposed to fibers, but not to asbestos fibers. However, exposure to asbestos fibers in the textile industry did occur in two small workshops in Sweden after the Second World War (Professor S. Krantz, personal communication). It may be that the asbestos-containing brake shoes in the textile machines caused a background level of airborne asbestos fibers in the machine halls, as was shown in an Italian non-asbestos textile industry [23]. Male textile workers showed no increase in risk, but their work activities and exposure may have been different.

It is well known that textile workers have an increased risk of mesothelioma, as was seen in the US [24] and several other countries where asbestos fibers have been a part of the production process. However, no studies have confirmed that exposure to textile fibers alone is carcinogenic. The traditional understanding of mesothelioma incidence in women holds that they are exposed via their husbands; that is, men exposed to asbestos bring home asbestos fibers on their clothes, and their wives are then subjected to secondary asbestos exposure when they handle or wash the clothes. However, we did not find any evidence of such a pattern in the present analysis.

Linkage to the Swedish NOCCA-JEM led to some interesting results. Twelve of the 17 occupations with excess mesothelioma risk were rated for asbestos exposure in the Swedish NOCCA-JEM. Five occupations (mechanical engineers and technicians; toolmakers, machine tool setters and operators; chemical process workers; paper pulp workers; and stationary engine and related equipment operators) were rated for chemical exposure in the Swedish NOCCA-JEM, but not for asbestos exposure. It is possible that asbestos exposure occurred in all of these occupations, but that the Swedish NOCCA-JEM is not sensitive enough to rate them as such due to low asbestos prevalence (e.g., a total of 102,447 men had the occupational code of mechanical engineers and technicians in 1980). Moreover, these occupations contain many different activities and tasks that include low asbestos exposure. We excluded all occupations in the Swedish NOCCA-JEM with an exposure prevalence (proportion of exposed individuals in a defined occupation) of less than 5% for all agents.

Asbestos use was highest in Sweden from 1965 to 1975. Due to a latency period of 30 to 40 years, we should not expect to observe many changes until the last of our three follow-up periods, during which we found an increased risk for sheet metal workers and electrical fitters.

The excess risk for women sewing workers in the first follow-up period may have been due to exposure during the 1930s and 1940s, for which we do not have information. The same point can also be made for the other occupations that showed decreasing trends.

An important observation is that the following occupations rated for asbestos exposure in the Swedish NOCCA-JEM showed no elevated risk in our data: chemical and cellulose processing work and store and warehouse workers. Furnace men, non-specific electrical and electronics workers, concrete and construction workers, glass formers and cutters, paper and paperboard workers, and chimney sweeps are all exposed to asbestos in the course of their work. Although they all showed an elevated risk of mesothelioma in our analysis, it was not statistically significant. An increased risk of mesothelioma has been observed in other studies among furnace men and chimney sweeps [13,25].

Male ships’ engineers, as well as divers and pipe layers, showed a significant excess risk of mesothelioma of the pleura, but in the Swedish NOCCA-JEM these occupations were not rated for any chemical exposure. Male ships’ engineers are more likely to be exposed to asbestos [26].

Among women, canning workers, sorting clerks and postal workers, and packers are not exposed to asbestos, but we found a significant excess risk of mesothelioma of the pleura and peritoneum in these occupations. We have no explanation for these observations. Women working as sewing workers and cleaners also showed a significant excess risk of mesothelioma of the pleura. These occupations do include exposure to textile fibers, but no specific textile fiber has been correlated to mesothelioma in the literature.

A total of 25.5% of the women with mesothelioma in the present study had mesothelioma of the peritoneum, compared to just 5.9% of the men. A change in the diagnostic procedures for peritoneal tumors in 1999 may indicate a possibility for misclassification before the year 2000 [27]. Several studies have shown that ovarian and gastrointestinal tumors may be misdiagnosed as mesothelioma of the peritoneum and vice versa [28-30]. The distinction between ovarian cancer and mesothelioma of the peritoneum may also be difficult to detect. A significant excess risk of ovarian cancer has been found among textile workers, graphic workers, packing workers, and workers in the telegraph and telephone industry (a subgroup of postal workers) in Sweden [31], corresponding to employment in both 1960 and 1970. This is in agreement with our observations of excess risk in this study, but no reports have described potential asbestos exposure within these occupations.

Asbestos is known to cause cancer in the wives and children of workers who are heavily exposed to asbestos [32,33]. This association may indicate that even household exposure may play a role in the development of asbestos-related cancers [34]. However, we have no indication as to why the women in the high-risk occupations observed in this study would have had higher levels of household exposure than women in other occupations.

Asbestos may have been a contaminant in talc, particularly before the mid-1970s [35]. Talc may be used for hygienic purposes or may be introduced to the women peritoneum through powder-coated gloves used in surgical operations [36]. Heller et al. [37] found asbestos fibers in the ovarian tissue of 69% of women with household asbestos exposure and in 35% of those with a history of asbestos exposure. No associations were found between ovarian cancer and occupational exposure to talc in a Norwegian study of pulp and paper employees [38]. In a review of occupational risk factors and ovarian cancer, Shen et al. [39] observed an excess risk of ovarian cancer associated with talc exposure.

Exposure assessment is a weakness in most epidemiological studies. The NOCCA data lack information on smoking habits, but smoking is not expected to affect the risk of mesothelioma [40]. The Swedish NOCCA cohort was analyzed in conjunction with the Swedish NOCCA-JEM, which contains quantitative exposure levels for 25 carcinogenic agents for each year between 1945 and 1994 for 96 different occupations. Never before has any linkage been made using such large and advanced materials.

We have chosen to present the results as they are, in an exploratory manner, and acknowledge that some of the SIRs may have been significant by chance.

We were not able to determine which time scale (age, period, or cohort) influenced the observed trends. This is a weakness in this study and similar studies using SIRs as risk estimates.

Until recently, mesothelioma incidence has increased each year in Sweden, and it is therefore important to investigate the context of this increase, as we do not currently have a full understanding of the situation. There is no reason to believe that asbestos is the only causative agent of mesothelioma, or that the disease will disappear over time due to reduced asbestos exposure. Therefore, it is important to identify other potential airborne risk factors that may be liabilities to the working population. The follow-up time after the asbestos ban in Sweden is too short (9-17 years in the latest follow-up period) to illustrate that effect.

## Figures and Tables

**Table 1.** Observed number of mesotheliomas in the peritoneum and pleura among men in Sweden and SIRs from 1961 to 2009

NYK	Occupational title, 1980	N	Peritoneum (ICD-7 158)			Pleura (ICD-7 162.2)		
			Obs	SIR	95% CI	Obs	SIR	95% CI
Occupations exposed to carcinogenic substances								
003	Mechanical engineers and technicians	102,447	6	1.08	0.40, 2.36	158	1.67	1.42, 1.95
631	Railway engine drivers and assistants	9,605	1	1.71	0.04, 9.54	19	1.82	1.09, 2.84
750	Toolmakers, machine-tool setters, and operators	95,837	7	1.43	0.57, 2.94	120	1.48	1.22, 1.77
751	Machinery fitters and machine assemblers	127,226	6	0.96	0.35, 2.10	180	1.75	1.50, 2.02
753	Sheet metal workers	38,172	3	1.55	0.32, 4.52	140	4.38	3.68, 5.17
754	Plumbers and pipe fitters	31,515	6	3.56	1.31, 7.76	139	4.99	4.20, 5.90
755	Welders and flame cutters	41,203	3	1.45	0.30, 4.24	54	1.57	1.18, 2.04
761	Electrical fitters and wiremen	63,493	7	2.21	0.89, 4.55	119	2.28	1.89, 2.72
771	Construction carpenters and joiners	83,073	2	0.46	0.06, 1.67	98	1.40	1.14, 1.70
781	Painters	49,244	7	2.70	1.08, 5.56	73	1.72	1.35, 2.16
791	Bricklayers	18,956	6	5.42	1.99, 11.80	29	1.60	1.07, 2.30
794	Insulators	2,589	8	64.70	28.0, 128.00	22	10.90	6.81, 16.50
799	Non-specified other building and construction work	7,333	0	0.00	0.00, 7.42	22	2.75	1.72, 4.17
831	Chemical process workers	8,783	0	0.00	0.00, 8.44	15	2.15	1.20, 3.54
834	Paper pulp workers	9,431	0	0.00	0.00, 7.29	17	2.10	1.22, 3.36
871	Stationary engine and related equipment operators	11,851	2	3.14	0.38, 11.30	18	1.77	1.05, 2.79
873	Riggers and cable splicers	269	0	0.00	0.00, 252.00	2	8.64	1.05, 31.20
Occupations unexposed to carcinogenic substances agents								
603	Ships’ engineers	3,895	1	5.32	0.13, 29.60	19	6.06	3.65, 9.46
797	Divers and pipe layers	2,983	0	0.00	0.00, 56.90	4	4.20	1.14, 10.70

SIR, standardized incidence ratio; NYK, Nordic Occupational Classification; N, number of persons in follow-up; ICD, International Classification of Diseases; Obs, observed; CI, confidence interval.

**Table 2.** Observed number of mesotheliomas in the peritoneum and pleura among women in Sweden and SIRs from 1961 to 2009

NYK	Occupational title, 1980	N	Peritoneum (ICD-7 158)			Pleura (ICD-7 162.2)		
			Obs	SIR	95% CI	Obs	SIR	95% CI
Occupations exposed to carcinogenic substances								
718	Sewing work n.e.c.	33,031	2	0.75	0.09, 2.70	18	2.14	1.27, 3.39
Occupations unexposed to carcinogenic substances								
661	Sorting clerks and postal workers	6,028	2	9.27	1.12, 33.50	1	1.47	0.04, 8.21
825	Canning workers	3,882	0	0.00	0.00, 13.40	4	4.90	1.33, 12.50
881	Packers	15,318	4	4.49	1.22, 11.50	3	1.07	0.22, 3.13
932	Cleaners	103,538	7	1.21	0.49, 2.49	27	1.59	1.05, 2.31

SIR, standardized incidence ratio; NYK, Nordic Occupational Classification; N, number of persons in follow-up; ICD, International Classification of Diseases; Obs, observed; CI, confidence interval; n.e.c., not elsewhere classified.

**Table 3.** Observed number of mesotheliomas of the pleura among men and women in Sweden and SIRs for three time periods; 1961-1974, 1975-1989, and 1990-2009

NYK	Occupational title	1961-1974			1975-1989			1990-2009		
		Obs	SIR	95% CI	Obs	SIR	95% CI	Obs	SIR	95% CI
Exposed to chemical agents, men										
003	Mechanical engineers and technicians	4	1.31	0.36, 3.37	42	1.85	1.34, 2.51	112	1.62	1.34, 1.95
750	Toolmakers, machine-tool setters and operators	4	1.16	0.32, 2.97	40	1.83	1.31, 2.49	76	1.36	1.07, 1.70
751	Machinery fitters and machine assemblers	10	2.67	1.28, 4.91	52	2.03	1.51, 2.66	118	1.60	1.33, 1.92
753	Sheet metal workers	4	2.75	0.75, 7.04	38	4.27	3.02, 5.86	98	4.53	3.68, 5.52
754	Plumbers and pipe fitters	5	4.12	1.34, 9.62	44	5.80	4.22, 7.79	90	4.73	3.80, 5.81
755	Welders and flame cutters	4	3.90	1.06, 9.98	15	1.83	1.02, 3.01	35	1.39	0.97, 1.93
761	Electrical fitters and wiremen	4	2.09	0.57, 5.35	27	2.13	1.40, 3.10	88	2.34	1.87, 2.88
771	Construction carpenters and joiners	3	0.66	0.14, 1.92	34	1.45	1.01, 2.03	61	1.45	1.11, 1.86
781	Painters	4	1.57	0.43, 4.01	20	1.46	0.89, 2.25	49	1.88	1.39, 2.48
794	Insulators	0	0.00	0.00, 42.10	7	12.81	5.15, 26.40	15	10.79	6.04, 17.80
799	Non-specified other building and construction work	0	0.00	0.00, 7.91	7	2.70	1.09, 5.57	15	3.04	1.70, 5.01
831	Chemical process workers	2	3.70	0.45, 13.40	3	1.18	0.24, 3.46	10	2.55	1.22, 4.69
871	Stationary engine and related equipment operators	5	5.87	1.91, 13.70	5	1.32	0.43, 3.08	8	1.44	0.62, 2.84
873	Riggers and cable splicers	0	0.00	0.00, 178.00	0	0.00	0.00, 41.8	2	16.33	1.98, 59.00
Unexposed to chemical agents, men										
603	Ships’ engineers	0	0.00	0.00, 23.60	7	7.88	3.17, 16.20	12	5.73	2.96, 10.00
797	Divers and pipelayers				0	0.00	0.00, 42.50	4	4.62	1.26, 11.80
Exposed to chemical agents, women										
718	Sewing work n.e.c.	4	5.65	1.54, 14.50	9	3.07	1.40, 5.83	5	1.05	0.34, 2.45

SIR, standardized incidence ratio; NYK, Nordic Occupational Classification; Obs, observed; CI, confidence interval; n.e.c., not elsewhere classified.

**Table 4.** Observed number of mesotheliomas of the peritoneum among men and women in Sweden and SIRs for three time periods; 1961-1974, 1975-1989, and 1990-2009

NYK	Occupational title	1961-1974			1975-1989			1990-2009		
		Obs	SIR	95% CI	Obs	SIR	95% CI	Obs	SIR	95% CI
Exposed to chemical agents, men										
754	Plumbers and pipe fitters	0	0.00	0.00, 30.60	2	3.11	0.38, 11.3	4	4.34	1.18, 11.10
761	Electrical fitters and wiremen	0	0.00	0.00, 19.70	1	0.93	0.02, 5.18	6	3.14	1.15, 6.85
791	Bricklayers	1	7.89	0.20, 43.90	3	5.85	1.21, 17.10	2	4.28	0.52, 15.50
794	Insulators	3	346.21	71.40, 1,012.00	3	65.27	13.50, 191.00	2	29.01	3.51, 105.00
Unexposed to chemical agents, women										
661	Sorting clerks and postal workers	0	0.00	0.00, 454.00	0	0.00	0.00, 85.20	2	12.16	1.47, 43.90

SIR, standardized incidence ratio; NYK, Nordic Occupational Classification; Obs, observed; CI, confidence interval; n.e.c., not elsewhere classified.

## Appendix 1.

Exposure codes, exposures, and units used in the Swedish job-exposure matrix

## Appendix 2.

Observed number of mesotheliomas in the peritoneum and pleura among men in Sweden in occupations exposed to chemical agents with SIRs from 1961 to 2009

## Appendix 3.

Observed number of mesotheliomas in the peritoneum and pleura among men in Sweden in occupations not exposed to chemical agents with SIRs from 1961 to 2009

## Appendix 4.

Observed number of mesotheliomas in the peritoneum and pleura among women in Sweden in occupations exposed to chemical agents with SIRs from 1961 to 2009

## Appendix 5.

Observed number of mesotheliomas in the peritoneum and pleura among women in Sweden in occupations not exposed to chemical agents with SIRs from 1961 to 2009
