# Supplementary material for: Occupation and mesothelioma in Sweden: updated incidence in men and women in the 27 years after the asbestos ban
Source: Epidemiol Health. 2016 Sep 20;38:e2016039. doi: 10.4178/epih.e2016039 (PMC5114438; doi:10.4178/epih.e2016039)
Supplement: Supplementary file 1 [file epih-38-e2016039-app1.pdf]

Appendix 1. Exposure codes, exposures, and units used in the Swedish job-exposure matrix

Exposure code	Exposure	Unit
ALHC	Aliphatic and alicyclic hydrocarbon solvents	ppm
ANIM	Animal-borne dust	mg/m^3
ARHC	Aromatic hydrocarbon solvents	ppm
ASB	Asbestos	f/cm^3
BAP	Benzo(a)pyrene	µg/m^3
BITU	Bitumen fumes	mg/m^3
CHC	Chlorinated hydrocarbon solvents	ppm
CR	Chromium	µg/m^3
DEEX	Diesel engine exhaust	mg/m^3
FE	Iron	mg/m^3
FORM	Formaldehyde	ppm
GASO	Gasoline	ppm
NI	Nickel	µg/m^3
OSOL	Other organic solvents	ppm
PB	Lead	µmol/L
QUAR	Quartz dust	mg/m^3
SO_2	Sulfur dioxide	ppm
WOOD	Wood dust	mg/m^3
WELD	Welding fumes	mg/m^3
BENZ	Benzene	ppm
MCH	Methylene chloride	ppm
TOLU	Toluene	ppm
PER	Perchloroethylene	ppm
TCE	1, 1, 1-trichloroethane	ppm
TRI	Trichloroethylene	ppm
UV	Ultraviolet radiation	J/m^2
IRAD	Ionizing radiation	mSv
PPWL	Perceived physical work load	score
NIGH	Night work	none
NOEXP	No exposure to any known carcinogens	none
