# Supplementary material for: Occupation and mesothelioma in Sweden: updated incidence in men and women in the 27 years after the asbestos ban
Source: Epidemiol Health. 2016 Sep 20;38:e2016039. doi: 10.4178/epih.e2016039 (PMC5114438; doi:10.4178/epih.e2016039)
Supplement: Supplementary file 2 [file epih-38-e2016039-app2.pdf]

Appendix 2. Observed number of mesotheliomas in the peritoneum and pleura among men in Sweden in occupations exposed to chemical agents with SIRs from 1961 to 2009

NYK	Occupational title, 1980	Exposure code[1]	N	Peritoneum (ICD-7 158)			Pleura (ICD-7 162.2)		
				Obs	SIR	95% CI	Obs	SIR	95% CI
003	Mechanical engineers and technicians	DEEX	102,447	6	1.08	0.40, 2.36	158	1.67	1.42, 1.95
011	Chemists and physicists	BENZ, NIGW	8,340	0	0.00	0.00, 9.14	2	0.30	0.04, 1.08
014	Laboratory technicians and assistants	BENZ	2,091	0	0.00	0.00, 78.60	1	1.34	0.03, 7.48
021	Veterinarians	ANIM, PPWL, UV	1,256	0	0.00	0.00, 53.10	0	0.00	0.00, 3.09
045	Medical technicians	FORM, NIGW	2,108	0	0.00	0.00, 29.40	4	2.01	0.55, 5.13
338	Filling station attendants and demonstrators	BENZ, DEEX, GASO, NIGW, PPWL, TOLU	8,248	2	3.89	0.47, 14.10	3	0.35	0.07, 1.03
401	Working proprietors; agricultural, horticultural, and forestry enterprises	ANIM, PPWL, UV	202,251	9	0.71	0.32, 1.34	49	0.23	0.17, 0.31
405	Livestock breeders	ANIM, FORM	1,194	0	0.00	0.00, 51.80	2	1.72	0.21, 6.21
406	Breeders of fur-bearing animals	ANIM, PPWL, UV	967	0	0.00	0.00, 59.00	0	0.00	0.00, 3.55
411	Agricultural and livestock workers	ANIM, PPWL, UV	52,282	1	0.31	0.01, 1.71	5	0.09	0.03, 0.22
414	Fur-bearing animal farm workers	ANIM, PPWL, UV	488	0	0.00	0.00, 113.00	0	0.00	0.00, 6.71
418	Agricultural, horticultural, and livestock work n.e.c.	ANIM, PPWL, UV	905	0	0.00	0.00, 93.20	1	1.54	0.04, 8.58
419	Non-specified agricultural, horticultural, and livestock work	ANIM, PPWL, UV	7	0	0.00	0.00, 8,212.00	0	0.00	0.00, 441.00
441	Forest workers and log-drivers	FORM, PPWL, UV	65,024	1	0.26	0.01, 1.46	14	0.22	0.12, 0.38
501	Miners and quarrymen	BAP, DEEX, IRAD, PPWL, QUAR	12,178	0	0.00	0.00, 5.57	5	0.47	0.15, 1.11
502	Well drillers and diamond drillers	PPWL, QUAR, UV	1,829	0	0.00	0.00, 36.10	2	1.20	0.15, 4.35
503	Ore dressers	NI, PPWL, QUAR	1,065	0	0.00	0.00, 64.40	1	1.11	0.03, 6.18
504	Mining and quarrying work n.e.c.	DEEX, QUAR	3,674	0	0.00	0.00, 19.20	2	0.65	0.08, 2.34
509	Non-specified mining and quarrying work	DEEX, QUAR	37	0	0.00	0.00, 1,710.00	0	0.00	0.00, 105.00
631	Railway engine drivers and assistants	ASB, DEEX, NIGW	9,605	1	1.71	0.04, 9.54	19	1.82	1.09, 2.84
633	Motor-vehicle drivers and tram drivers	DEEX, NIGW	155,917	7	0.87	0.35, 1.79	76	0.57	0.45, 0.71
635	Deliverymen	DEEX, NIGW, PPWL, UV	2,214	0	0.00	0.00, 46.70	0	0.00	0.00, 3.16
636	Bus and tram conductors, traffic assistants	DEEX, NIGW, UV	1,439	0	0.00	0.00, 48.00	0	0.00	0.00, 2.79
639	Non-specified rail and road transport work	DEEX, NIGW, UV	1,417	0	0.00	0.00, 53.40	4	3.61	0.98, 9.25
641	Harbormasters	DEEX, NIGW	958	0	0.00	0.00, 75.30	0	0.00	0.00, 4.63
644	Road traffic supervisors	DEEX, NIGW	6,829	0	0.00	0.00, 11.20	2	0.37	0.04, 1.34
649	Non-specified traffic supervision	DEEX, NIGW	5	0	0.00	0.00, 11,844.00	0	0.00	0.00, 589.00
712	Fur tailors	ANIM	1,074	0	0.00	0.00, 54.50	1	0.86	0.02, 4.80
714	Upholsterers	BENZ, MCH, PPWL, TCE, TOLU, WOOD	6,569	1	2.62	0.07, 14.60	5	0.78	0.25, 1.82
715	Patternmakers and cutters	FORM	2,294	0	0.00	0.00, 24.00	2	0.76	0.09, 2.75
718	Sewing work n.e.c.	FORM	1,563	0	0.00	0.00, 42.00	3	2.06	0.43, 6.03
722	Shoe cutters, lasters, and sewers	BENZ, TOLU	3,642	0	0.00	0.00, 17.30	1	0.28	0.01, 1.58
729	Non-specified shoe and leather goods work	BENZ, TOLU	10	0	0.00	0.00, 6,035.00	0	0.00	0.00, 323.00
731	Furnacemen	ASB, BAP, CR, FE, NI, NIGW, PB, PPWL, QUAR, SO2	11,199	1	1.74	0.04, 9.72	6	0.65	0.24, 1.42
732	Metal annealers, temperers, and case-hardeners	BAP, CR, FE, NI, NIGW, PB, PPWL, WELD	2,586	0	0.00	0.00, 26.40	2	0.89	0.11, 3.22
733	Rolling mill workers	CR, CR, FE, NI, NI, NIGW, PPWL, SO2, WELD	5,788	0	0.00	0.00, 11.50	4	0.76	0.21, 1.96

(Continued to the next page)

Appendix 2. Continued

NYK	Occupational title, 1980	Exposure code[1]	N	Peritoneum (ICD-7 158)			Pleura (ICD-7 162.2)		
				Obs	SIR	95% CI	Obs	SIR	95% CI
735	Smiths and forgers	BAP, FE, NIGW, PB, PPWL, WELD	10,725	1	1.68	0.04, 9.37	13	1.34	0.72, 2.30
736	Metal casters and moulders	BAP, CR, FE, FORM, NI, NIGW, PB, PPWL, QUAR, WELD	11,315	0	0.00	0.00, 5.73	10	0.96	0.46, 1.76
737	Wire and tube drawers	CR, FE, NI, NIGW, PPWL, WELD	2,594	0	0.00	0.00, 25.90	1	0.43	0.01, 2.40
738	Metal processing work n.e.c.	CR, FE, NI, PB, PPWL, QUAR, SO2, TCE, TRI, WELD	6,940	1	2.78	0.07, 15.50	10	1.73	0.83, 3.19
739	Non-specified metal processing work	CR, FE, NI, PB, PPWL, QUAR, SO2, TCE, TRI, WELD	503	0	0.00	0.00, 187.00	1	2.76	0.07, 15.40
750	Toolmakers, machine-tool setters, and operators	BAP, CR, FE, NI, PB, PPWL, TCE, TRI, WELD	95,837	7	1.43	0.57, 2.94	120	1.48	1.22, 1.77
751	Machinery fitters and machine assemblers	ASB, BAP, BENZ, CR, DEEX, FE, GASO, MCH, NI, PB, PPWL, TCE, TOLU, WELD	127,226	6	0.96	0.35, 2.10	180	1.75	1.50, 2.02
753	Sheet metal workers	ASB, CR, FE, NI, PB, PPWL, TRI, UV, WELD	38,172	3	1.55	0.32, 4.52	140	4.38	3.68, 5.17
754	Plumbers and pipe fitters	ASB, CR, FE, NI, PB, PPWL, WELD	31,515	6	3.56	1.31, 7.76	139	4.99	4.20, 5.90
755	Welders and flame cutters	ASB, BAP, CR, FE, FORM, NI, PB, PPWL, UV, WELD	41,203	3	1.45	0.30, 4.24	54	1.57	1.18, 2.04
757	Metal platers and coaters	CR, FE, FORM, NI, PB, PER, PPWL, TCE, TRI, WELD	3,090	0	0.00	0.00, 23.00	6	2.32	0.85, 5.05
759	Non-specified engineering and building metal work	CR, FE, NI, PB, PPWL, TCE, TRI, WELD	21,302	0	0.00	0.00, 2.66	20	0.87	0.53, 1.34
761	Electrical fitters and wiremen	ASB, PPWL	63,493	7	2.21	0.89, 4.55	119	2.28	1.89, 2.72
764	Radio and television assemblers and repairmen	MCH, PB, PER, TCE, TRI	15,557	1	1.39	0.04, 7.73	12	1.00	0.52, 1.75
766	Telephone and telegraph installers and repairmen	PB, PPWL, UV	21,069	0	0.00	0.00, 3.43	8	0.44	0.19, 0.86
769	Non-specified electrical and electronics work	ASB, FORM, MCH, PB, PER, TCE, TRI	4,649	1	3.35	0.08, 18.60	6	1.21	0.44, 2.63
771	Construction carpenters and joiners	ASB, PPWL, QUAR, UV, WOOD	83,073	2	0.46	0.06, 1.67	98	1.40	1.14, 1.70
772	Bench carpenters and cabinet makers	FORM, PPWL, WOOD	47,242	2	0.76	0.09, 2.75	54	1.25	0.94, 1.63
773	Laminated wood and fiberboard workers	CR, FORM, PPWL, WOOD	543	0	0.00	0.00, 334.00	1	6.26	0.16, 34.90
774	Frame and circular sawyers and planers	CR, NIGW, PPWL, WOOD	11,368	0	0.00	0.00, 6.57	3	0.33	0.07, 0.98
778	Woodwork n.e.c.	PPWL, WOOD	9,787	1	1.72	0.04, 9.58	8	0.85	0.37, 1.67
779	Non-specified woodwork	PPWL, WOOD	8,632	0	0.00	0.00, 6.72	4	0.45	0.12, 1.16
781	Painters	ASB, BENZ, CR, FORM, MCH, PB, PPWL, TCE, TOLU, UV	49,244	7	2.70	1.08, 5.56	73	1.72	1.35, 2.16
791	Bricklayers	ASB, PPWL, QUAR, UV	18,956	6	5.42	1.99, 11.80	29	1.60	1.07, 2.30
792	Masons	PPWL, QUAR, UV	252	0	0.00	0.00, 267.00	1	4.52	0.11, 25.20
793	Concrete and construction workers	ASB, PPWL, QUAR, UV	72,332	5	1.29	0.42, 3.01	76	1.23	0.97, 1.54
794	Insulators	ASB, BAP, BITU, PPWL, UV	2,589	8	64.70	28.0, 128.00	22	10.90	6.81, 16.50
799	Non-specified other building and construction work	ASB, PB, PPWL, QUAR, UV	7,333	0	0.00	0.00, 7.42	22	2.75	1.72, 4.17
801	Typographers and lithographers	PB	27,145	1	0.72	0.02, 4.00	13	0.56	0.30, 0.97
806	Bookbinders	MCH, PPWL	2,842	0	0.00	0.00, 25.00	0	0.00	0.00, 1.54
808	Printing work n.e.c.	BENZ, GASO, MCH, PB, PPWL, TCE, TOLU	652	0	0.00	0.00, 150.00	1	2.72	0.07, 15.10
809	Non-specified printing work	BENZ, GASO, PB, TOLU, TRI	15	0	0.00	0.00, 4,343.00	0	0.00	0.00, 247.00
811	Glass formers and cutters	ASB, NI, NIGW, PB, PPWL, QUAR	2,083	0	0.00	0.00, 30.00	3	1.47	0.30, 4.30
812	Potters	NI, NIGW, PB, PPWL, QUAR	1,758	0	0.00	0.00, 36.40	0	0.00	0.00, 2.22

(Continued to the next page)

Appendix 2. Continued

NYK	Occupational title, 1980	Exposure code[1]	N	Peritoneum (ICD-7 158)			Pleura (ICD-7 162.2)		
				Obs	SIR	95% CI	Obs	SIR	95% CI
813	Glass and ceramics kilnmen	NI, NIGW, PB, PPWL, QUAR, SO2	1,313	0	0.00	0.00, 48.10	2	1.66	0.20, 6.00
814	Glass, china, and ceramics painters and decorators	NI, NIGW, PB, QUAR	348	0	0.00	0.00, 188.00	0	0.00	0.00, 11.30
818	Glass, pottery, and tile work n.e.c.	NIGW, QUAR	1,691	0	0.00	0.00, 47.60	2	1.61	0.19, 5.80
819	Non-specified glass, pottery, and tile work	NIGW, QUAR	3,007	0	0.00	0.00, 20.40	4	1.38	0.38, 3.54
831	Chemical process workers	BENZ, MCH, NIGW, PER, PPWL, TOLU, TRI	8,783	0	0.00	0.00, 8.44	15	2.15	1.20, 3.54
834	Paper pulp workers	NIGW, SO2	9,431	0	0.00	0.00, 7.29	17	2.10	1.22, 3.36
836	Paper and paperboard workers	ASB, FORM, NIGW, PPWL	15,354	1	1.29	0.03, 7.17	13	1.04	0.56, 1.79
838	Chemical and cellulose processing work n.e.c.	ASB, BENZ, BITU, FORM, MCH, NIGW, PB, PPWL, QUAR, TOLU	2,050	0	0.00	0.00, 34.9	1	0.59	0.01, 3.29
839	Non-specified chemical and cellulose processing work	NIGW, SO2	4,724	0	0.00	0.00, 14.90	3	0.77	0.16, 2.24
851	Rubber products workers	BENZ, NIGW, PPWL, TCE, TOLU, TRI	7,891	1	2.46	0.06, 13.70	9	1.36	0.62, 2.59
852	Plastic products workers	FORM, MCH, NIGW, PB, PPWL, TCE	7,628	0	0.00	0.00, 10.70	8	1.47	0.63, 2.89
853	Tanners and fur dressers	CR, NIGW, PPWL	1,545	0	0.00	0.00, 39.80	1	0.65	0.02, 3.62
856	Stone cutters and carvers	NIGW, PPWL, QUAR	3,223	0	0.00	0.00, 20.70	2	0.72	0.09, 2.60
857	Paper and paperboard products workers	FORM, NIGW, PPWL	3,920	1	5.42	0.14, 30.20	2	0.66	0.08, 2.39
871	Stationary engine and related equipment operators	DEEX, NIGW, SO2	11,851	2	3.14	0.38, 11.30	18	1.77	1.05, 2.79
872	Crane and hoist operators	ASB, NIGW	9,881	0	0.00	0.00, 6.56	14	1.51	0.83, 2.54
873	Riggers and cable splicers	ASB, NI, PPWL	269	0	0.00	0.00, 252.00	2	8.64	1.05, 31.2
874	Construction machine operators	BITU, DEEX, PPWL	24,172	1	0.77	0.02, 4.27	8	0.37	0.16, 0.72
875	Truck and conveyor operators	DEEX, NIGW, PPWL	27,549	2	1.54	0.19, 5.56	14	0.67	0.37, 1.13
876	Greasers	DEEX, PPWL, TOLU	3,077	0	0.00	0.00, 22.90	3	1.16	0.24, 3.40
879	Non-specified operations monitoring and material handling work	DEEX, PPWL, TOLU	60	0	0.00	0.00, 1,562.00	0	0.00	0.00, 110.00
882	Dockers and freight handlers	DEEX, PPWL, UV	13,522	1	1.39	0.04, 7.77	9	0.79	0.36, 1.51
883	Store and warehouse workers	ASB, PPWL	59,447	0	0.00	0.00, 1.25	45	0.96	0.70, 1.29
902	Policemen	DEEX, NIGW, PB, PPWL	20,783	1	0.92	0.02, 5.12	17	0.92	0.54, 1.47
933	Chimney sweeps	ASB, BAP, PPWL	2,656	0	0.00	0.00, 27.70	5	2.29	0.74, 5.34
943	Launderers and dry-cleaners	BENZ, PER, PPWL, TCE, TRI	3,130	0	0.00	0.00, 21.90	3	1.09	0.22, 3.18

SIR, standardized incidence ratio; NYK, Nordic Occupational Classification; N, number of persons in follow-up; ICD, International Classification of Diseases; Obs, observed; CI, confidence interval; n.e.c., not elsewhere classified.
[1]See Appendix 1 for exposure codes.
