# Supplementary material for: Occupation and mesothelioma in Sweden: updated incidence in men and women in the 27 years after the asbestos ban
Source: Epidemiol Health. 2016 Sep 20;38:e2016039. doi: 10.4178/epih.e2016039 (PMC5114438; doi:10.4178/epih.e2016039)
Supplement: Supplementary file 3 [file epih-38-e2016039-app3.pdf]

Appendix 3. Observed number of mesotheliomas in the peritoneum and pleura among men in Sweden in occupations not exposed to chemical agents with SIRs from 1961 to 2009

NYK	Occupation title, 1980	Exposure code[1]	N	Peritoneum (ICD-7 158)			Pleura (ICD-7 162.2)		
				Obs	SIR	95% CI	Obs	SIR	95% CI
001	Architects, building and construction engineers and technicians	NOEXP	70,114	5	1.30	0.42, 3.03	41	0.62	0.45, 0.84
002	Electrical, electronics, and telecommunications engineers and technicians	NOEXP	58,669	1	0.37	0.01, 2.05	54	1.19	0.89, 1.55
004	Chemical engineers and technicians	NOEXP	20,932	1	0.93	0.02, 5.18	24	1.33	0.85, 1.99
005	Metallurgists and mining engineers and technicians	NOEXP	9,376	1	1.97	0.05, 11.00	6	0.70	0.26, 1.51
006	Engineers and technicians n.e.c.	NOEXP	28,910	0	0.00	0.00, 2.36	27	1.03	0.68, 1.50
007	Surveyors, measurers, and cartographers	UV	5,085	0	0.00	0.00, 15.30	1	0.24	0.01, 1.33
008	Technical assistants	NOEXP	4,115	0	0.00	0.00, 14.00	5	1.09	0.35, 2.55
009	Non-specified engineering work	NOEXP	4,919	0	0.00	0.00, 30.80	0	0.00	0.00, 2.17
013	Geologists and meteorologists	UV	1,722	0	0.00	0.00, 46.70	1	0.73	0.02, 4.04
019	Non-specified chemical and physical work	NOEXP	2,109	0	0.00	0.00, 22.90	3	1.06	0.22, 3.11
022	Biologists	UV	813	0	0.00	0.00, 137.00	0	0.00	0.00, 8.98
023	Agricultural and horticultural researchers/advisors	UV	3,363	1	5.48	0.14, 30.50	1	0.32	0.01, 1.80
024	Forestry researchers/advisors	PPWL, UV	1,865	0	0.00	0.00, 35.40	0	0.00	0.00, 2.15
029	Non-specified biological work	UV	3	0	0.00	0.00, 21,907.00	0	0.00	0.00, 1,096.00
031	Physicians and surgeons	NOEXP	19,366	0	0.00	0.00, 4.51	5	0.37	0.12, 0.86
032	Dentists	NOEXP	7,386	0	0.00	0.00, 9.73	1	0.16	0.00, 0.87
039	Non-specified medical work	NIGW, PPWL	2	0	0.00	0.00, 19,278.00	0	0.00	0.00, 1,044.00
040	Registered nurses	NIGW, PPWL	3,526	0	0.00	0.00, 49.00	1	1.12	0.03, 6.22
041	Midwives	NIGW, PPWL	24	0	0.00	0.00, 6,856.00	0	0.00	0.00, 458.00
042	Attendants in psychiatric care	NIGW	8,595	2	5.14	0.62, 18.60	1	0.16	0.00, 0.87
043	Practical nurses and hospital orderlies	NIGW, PPWL	7,382	0	0.00	0.00, 20.20	2	0.82	0.10, 2.97
044	Dental nurses	NOEXP	29	0	0.00	0.00, 5,239.00	0	0.00	0.00, 375.00
046	Pharmacists	NIGW	1,204	0	0.00	0.00, 53.40	0	0.00	0.00, 3.11
047	Physiotherapists and occupational therapists	PPWL	2,874	0	0.00	0.00, 37.40	1	0.67	0.02, 3.76
048	Health and nursing work n.e.c.	NIGW, PPWL	1,939	0	0.00	0.00, 46.80	4	3.24	0.88, 8.31
049	Non-specified health and nursing work	NIGW, PPWL	12	0	0.00	0.00, 14,649.00	0	0.00	0.00, 667.00
050	Principals and headmasters	NOEXP	4,649	0	0.00	0.00, 12.70	5	1.00	0.32, 2.33
051	University and higher education teachers	NOEXP	11,588	0	0.00	0.00, 7.61	6	0.73	0.27, 1.59
052	Teachers of theoretical subjects	PPWL	26,139	0	0.00	0.00, 2.68	7	0.30	0.12, 0.61
053	Schoolmasters	PPWL	19,143	2	1.98	0.24, 7.17	4	0.23	0.06, 0.58
054	Teachers of painting, music, and physical education	PPWL	11,770	1	1.80	0.05, 10.00	5	0.54	0.18, 1.26
055	Teachers of vocational subjects	PPWL	10,747	0	0.00	0.00, 5.90	13	1.21	0.65, 2.08
056	Pre-primary education teachers	PPWL, UV	1,788	0	0.00	0.00, 101.00	0	0.00	0.00, 8.63
057	Educational methods advisors	NOEXP	3,650	0	0.00	0.00, 17.80	0	0.00	0.00, 1.05
058	Educational work n.e.c.	NOEXP	1,849	0	0.00	0.00, 65.00	2	2.14	0.26, 7.72
059	Non-specified educational work	NOEXP	388	0	0.00	0.00, 398.00	0	0.00	0.00, 29.80
061	Ministers and priests	NOEXP	8,843	0	0.00	0.00, 7.41	3	0.36	0.07, 1.05
068	Religious work n.e.c.	NOEXP	575	0	0.00	0.00, 168.00	0	0.00	0.00, 10.40

(Continued to the next page)

Appendix 3. Continued

NYK	Occupation title, 1980	Exposure code[1]	N	Peritoneum (ICD-7 158)			Pleura (ICD-7 162.2)		
				Obs	SIR	95% CI	Obs	SIR	95% CI
069	Non-specified religious work	NOEXP	5	0	0.00	0.00, 14.349.00	0	0.00	0.00, 729.00
071	Judges and other lawyers in courts of law	NOEXP	2,674	0	0.00	0.00, 25.50	1	0.39	0.01, 2.17
072	Prosecutors and senior police officers	NOEXP	1,609	0	0.00	0.00, 39.60	1	0.63	0.02, 3.51
073	Lawyers in private practice	NOEXP	3,163	0	0.00	0.00, 27.10	1	0.43	0.01, 2.42
074	Corporation and organization lawyers	NOEXP	2,489	0	0.00	0.00, 36.50	0	0.00	0.00, 2.23
078	Legal work n.e.c.	NOEXP	72	0	0.00	0.00, 803.00	0	0.00	0.00, 44.10
079	Non-specified legal work	NOEXP	33	0	0.00	0.00, 1,702.00	0	0.00	0.00, 93.30
081	Sculptors, painters, photographers, and commercial artists	NOEXP	7,264	0	0.00	0.00, 10.30	5	0.83	0.27, 1.94
082	Designers	NOEXP	2,152	0	0.00	0.00, 33.00	3	1.57	0.32, 4.58
083	Display artists	NOEXP	1,773	0	0.00	0.00, 35.10	1	0.55	0.01, 3.08
084	Authors	NOEXP	717	0	0.00	0.00, 112.00	1	1.89	0.05, 10.50
085	Journalists and editors	NOEXP	10,784	1	2.08	0.05, 11.60	1	0.13	0.00, 0.71
086	Performing artists	NOEXP	1,785	0	0.00	0.00, 49.80	1	0.82	0.02, 4.58
087	Composers and musicians	NOEXP	5,853	0	0.00	0.00, 14.70	2	0.50	0.06, 1.80
088	Literary and artistic work n.e.c.	NOEXP	2,119	0	0.00	0.00, 41.20	0	0.00	0.00, 2.47
089	Non-specified literary and artistic work	NOEXP	112	0	0.00	0.00, 550.00	0	0.00	0.00, 32.40
091	Accountants and auditors	NIGW	7,763	0	0.00	0.00, 10.50	1	0.17	0.00, 0.96
092	Social workers	NIGW	11,545	0	0.00	0.00, 9.27	6	0.99	0.36, 2.15
093	Librarians, archivists, and curators	NOEXP	3,809	0	0.00	0.00, 21.90	0	0.00	0.00, 1.37
094	Economists and statisticians	NOEXP	9,809	1	3.69	0.09, 20.50	2	0.50	0.06, 1.80
095	Psychologists	NIGW	6,734	0	0.00	0.00, 9.40	6	0.89	0.33, 1.94
096	Staff officers	NOEXP	10,495	0	0.00	0.00, 9.26	1	0.15	0.00, 0.85
097	Systems analysts and programmers	NIGW	20,684	2	3.58	0.43, 12.90	6	0.75	0.27, 1.62
098	Other related work	NOEXP	2,464	0	0.00	0.00, 39.40	0	0.00	0.00, 2.44
099	Non-specified other professional, technical, and related work	NOEXP	105	0	0.00	0.00, 1,186.00	0	0.00	0.00, 55.80
101	Government legislative and administrative work	NOEXP	20,680	1	1.00	0.03, 5.57	10	0.57	0.27, 1.05
111	General managers	NOEXP	43,157	3	1.23	0.25, 3.59	28	0.69	0.46, 0.99
118	Other business managers, including managers with specific functions	NOEXP	42,128	0	0.00	0.00, 1.62	18	0.47	0.28, 0.74
119	Non-specified business administrative and other technical and economical administrative work	NOEXP	9	0	0.00	0.00, 6,826.00	0	0.00	0.00, 427.00
201	Bookkeepers and office cashiers	NOEXP	20,197	0	0.00	0.00, 3.12	11	0.55	0.27, 0.98
203	Bank tellers	NOEXP	551	0	0.00	0.00, 113.00	0	0.00	0.00, 6.86
204	Cashiers in retail stores and restaurants	PPWL	405	0	0.00	0.00, 206.00	0	0.00	0.00, 12.10
208	Debt collectors	NOEXP	908	1	23.77	0.60, 132.00	0	0.00	0.00, 5.19
209	Non-specified bookkeeping and clerical work	NOEXP	30	0	0.00	0.00, 2,087.00	0	0.00	0.00, 112.00
290	Secretaries, typists and related workers	NOEXP	13,491	1	2.26	0.06, 12.60	7	0.92	0.37, 1.90
291	Computer operators	NIGW	4,220	0	0.00	0.00, 23.00	2	0.78	0.09, 2.82
292	Bank employees (general bank work)	NOEXP	8,744	1	2.80	0.07, 15.60	3	0.51	0.11, 1.50
293	Travel agency employees	NOEXP	1,765	0	0.00	0.00, 44.50	1	0.71	0.02, 3.97

(Continued to the next page)

Appendix 3. Continued

NYK	Occupation title, 1980	Exposure code[1]	N	Peritoneum (ICD-7 158)			Pleura (ICD-7 162.2)		
				Obs	SIR	95% CI	Obs	SIR	95% CI
294	Forwarding and shipping agents.	NOEXP	7,092	0	0.00	0.00, 10.40	5	0.85	0.28, 1.98
295	Property managers and store managers	NOEXP	26,687	1	0.68	0.02, 3.77	23	0.95	0.60, 1.42
296	Insurance raters and claims adjusters	NOEXP	4,618	0	0.00	0.00, 15.70	2	0.51	0.06, 1.85
297	Employees in national insurance offices	NOEXP	2,571	0	0.00	0.00, 35.30	0	0.00	0.00, 2.26
298	Cost accountants and estimating clerks	NOEXP	18,612	0	0.00	0.00, 3.15	17	0.84	0.49, 1.35
299	Non-specified clerical work	NOEXP	10,738	2	2.58	0.31, 9.33	3	0.23	0.05, 0.68
301	Working proprietors, wholesale trade	NOEXP	10,565	1	1.97	0.05, 11.00	4	0.50	0.14, 1.28
302	Working proprietors, retail trade	NOEXP	44,588	2	0.82	0.10, 2.95	18	0.45	0.27, 0.71
309	Non-specified working proprietors	NOEXP	261	0	0.00	0.00, 448.00	1	9.44	0.24, 52.60
311	Insurance representatives and agents	NOEXP	4,736	0	0.00	0.00, 14.40	2	0.47	0.06, 1.69
312	Brokers and valuers	NOEXP	3,414	0	0.00	0.00, 28.20	1	0.49	0.01, 2.74
313	Advertising salesmen	NOEXP	11,279	0	0.00	0.00, 8.30	1	0.14	0.00, 0.77
319	Non-specified sales of insurance, real estate, securities, and business services	NOEXP	3	0	0.00	0.00, 215,113.00	0	0.00	0.00, 12,553.00
331	Commercial travelers, buyers, and dealers	NOEXP	106,733	6	1.16	0.42, 2.52	52	0.60	0.45, 0.78
332	Shop managers	PPWL	21,663	0	0.00	0.00, 2.87	13	0.60	0.32, 1.02
333	Shop assistants	PPWL	35,320	0	0.00	0.00, 1.98	22	0.71	0.45, 1.08
339	Non-specified other sales work	NOEXP	127	0	0.00	0.00, 604.00	0	0.00	0.00, 36.40
402	Farm managers and supervisors	PPWL, UV	2,381	0	0.00	0.00, 26.80	0	0.00	0.00, 1.55
403	Forestry managers and supervisors	UV	10,523	0	0.00	0.00, 5.38	1	0.09	0.00, 0.48
404	Horticultural managers and supervisors	NIGW, UV	3,170	0	0.00	0.00, 20.40	1	0.33	0.01, 1.85
407	Reindeer owners	PPWL, UV	807	0	0.00	0.00, 75.50	1	1.25	0.03, 6.94
409	Non-specified agricultural, horticultural, and forestry management work	PPWL, UV	13	0	0.00	0.00, 5,746.00	0	0.00	0.00, 306.00
412	Horticultural workers	PPWL, UV	17,272	0	0.00	0.00, 4.18	9	0.64	0.29, 1.22
415	Reindeer herdsmen	UV	253	0	0.00	0.00, 295.00	0	0.00	0.00, 17.20
421	Game-keepers and hunters	UV	208	0	0.00	0.00, 313.00	0	0.00	0.00, 17.70
431	Fishermen	PPWL, UV	9,029	0	0.00	0.00, 6.92	3	0.34	0.07, 1.01
432	Fish-breeders	PPWL, UV	231	0	0.00	0.00, 400.00	0	0.00	0.00, 28.00
601	Ships' deck officers	NIGW, UV	6,790	1	2.88	0.07, 16.10	8	1.38	0.60, 2.73
602	Ships' pilots	NIGW, UV	861	0	0.00	0.00, 67.70	1	1.04	0.03, 5.77
603	Ships' engineers	NIGW, UV	3,895	1	5.32	0.13, 29.60	19	6.06	3.65, 9.46
609	Non-specified ships' officers	NIGW, UV	6	0	0.00	0.00, 17,936.00	0	0.00	0.00, 1,012.00
611	Ships' deck and engine-room crew	NIGW, PPWL, UV	7,036	1	3.14	0.08, 17.50	10	1.96	0.94, 3.61
621	Aircraft pilots, navigators, and flight engineers	IRAD, NIGW	2,050	0	0.00	0.00, 38.90	0	0.00	0.00, 2.23
632	Railway guards	NIGW, UV	15,904	3	2.92	0.60, 8.53	11	0.60	0.30, 1.08
642	Air traffic controllers and flight dispatchers	NIGW	1,607	0	0.00	0.00, 59.20	0	0.00	0.00, 3.52
643	Railway station masters and train dispatchers	NIGW	4,799	1	3.30	0.08, 18.40	3	0.59	0.12, 1.74
651	Post-office clerks	NOEXP	5,220	0	0.00	0.00, 13.00	4	0.83	0.23, 2.12
652	Telecommunications traffic officers	NOEXP	362	0	0.00	0.00, 149.00	0	0.00	0.00, 8.49

(Continued to the next page)

Appendix 3. Continued

NYK	Occupation title, 1980	Exposure code[1]	N	Peritoneum (ICD-7 158)			Pleura (ICD-7 162.2)		
				Obs	SIR	95% CI	Obs	SIR	95% CI
653	Telephone operators	NOEXP	415	0	0.00	0.00, 221.00	0	0.00	0.00, 15.40
655	Telegraph and radio operators	NOEXP	1,358	0	0.00	0.00, 50.60	0	0.00	0.00, 2.89
659	Non-specified post and telecommunications work	NOEXP	4	0	0.00	0.00, 11,832.00	0	0.00	0.00, 613.00
661	Sorting clerks and postal workers	NIGW, PPWL, UV	19,489	1	1.10	0.03, 6.15	6	0.40	0.15, 0.88
662	Messengers	NIGW, PPWL, UV	9,941	1	2.45	0.06, 13.60	1	0.16	0.00, 0.90
669	Non-specified mail distribution and other messenger work	NIGW, PPWL, UV	9	0	0.00	0.00, 6,102.00	0	0.00	0.00, 509.00
671	Lighthouse and lock operators, ferry and harbor service assistants	NIGW, UV	1,406	0	0.00	0.00, 46.50	1	0.78	0.02, 4.33
678	Railway linemen	NIGW, UV	4,137	0	0.00	0.00, 17.50	1	0.30	0.01, 1.66
699	Non-specified transport and communications work	NIGW, UV	926	0	0.00	0.00, 58.20	1	0.92	0.02, 5.15
701	Spinners, weavers, knitters, and dyers	NIGW	12,935	2	2.76	0.33, 9.97	14	1.18	0.65, 1.98
711	Tailors and dressmakers	NOEXP	3,848	0	0.00	0.00, 16.80	1	0.29	0.01, 1.61
713	Milliners and hat makers	NOEXP	222	0	0.00	0.00, 273.00	0	0.00	0.00, 15.70
719	Non-specified sewing work	NIGW	280	0	0.00	0.00, 221.00	0	0.00	0.00, 13.40
721	Shoemakers and shoe repairers	NOEXP	4,100	0	0.00	0.00, 18.20	1	0.32	0.01, 1.80
726	Leather goods makers	NOEXP	1,506	0	0.00	0.00, 40.60	0	0.00	0.00, 2.43
741	Precision-tool makers	NIGW	8,077	0	0.00	0.00, 9.48	5	0.78	0.25, 1.81
742	Watchmakers	NOEXP	2,376	0	0.00	0.00, 26.00	0	0.00	0.00, 1.56
743	Opticians	NOEXP	1,570	0	0.00	0.00, 49.30	0	0.00	0.00, 2.98
744	Dental technicians	NOEXP	2,535	0	0.00	0.00, 29.00	2	0.94	0.11, 3.41
745	Goldsmiths and silversmiths	NOEXP	2,206	0	0.00	0.00, 29.60	3	1.41	0.29, 4.13
749	Non-specified precision-tool manufacturing work	NOEXP	30	0	0.00	0.00, 2,012.00	0	0.00	0.00, 112.00
765	Recording, sound, and light equipment operators	NOEXP	1,515	0	0.00	0.00, 113.00	0	0.00	0.00, 7.71
795	Glaziers	PPWL, UV	3,693	0	0.00	0.00, 19.90	6	1.97	0.72, 4.29
797	Divers and pipe layers	PPWL	2,983	0	0.00	0.00, 56.90	4	4.20	1.14, 10.70
821	Grain mill and oil press workers	PPWL	2,668	0	0.00	0.00, 24.40	0	0.00	0.00, 1.51
822	Bakers and pastry cooks	PPWL	15,529	0	0.00	0.00, 4.08	9	0.59	0.27, 1.13
823	Chocolate and confectionary workers	PPWL	958	0	0.00	0.00, 82.20	0	0.00	0.00, 5.18
824	Brewery, distillery, and other beverage plant workers	PPWL	2,361	0	0.00	0.00, 31.40	2	1.10	0.13, 3.97
825	Canning workers	PPWL	1,746	0	0.00	0.00, 39.70	1	0.67	0.02, 3.75
826	Butchers and meat preparers	PPWL	11,550	0	0.00	0.00, 6.11	2	0.20	0.02, 0.74
827	Dairy workers	PPWL	5,108	0	0.00	0.00, 12.50	1	0.20	0.01, 1.13
828	Food processing work n.e.c.	PPWL	1,677	0	0.00	0.00, 49.20	2	1.65	0.20, 5.97
829	Non-specified food processing work	PPWL	30	0	0.00	0.00, 2,155.00	0	0.00	0.00, 134.00
841	Tobacco workers	NIGW, PPWL	219	0	0.00	0.00, 358.00	0	0.00	0.00, 21.90
850	Basketry weavers	NIGW	331	0	0.00	0.00, 186.00	0	0.00	0.00, 11.70
854	Photographic laboratory workers	PPWL	704	0	0.00	0.00, 122.00	0	0.00	0.00, 7.71
855	Musical instrument makers and tuners	NIGW	812	0	0.00	0.00, 81.80	1	1.32	0.03, 7.36
858	Other production and related work n.e.c.	PPWL	12,425	1	1.51	0.04, 8.43	18	1.67	0.99, 2.64
859	Non-specified other production and related work	PPWL	6	0	0.00	0.00, 34,047.00	0	0.00	0.00, 1,939.00
861	Unskilled manual workers	PPWL	61,982	1	0.29	0.01, 1.64.00	45	0.83	0.60, 1.11

(Continued to the next page)

Appendix 3. Continued

NYK	Occupation title, 1980	Exposure code[1]	N	Peritoneum (ICD-7 158)			Pleura (ICD-7 162.2)		
				Obs	SIR	95% CI	Obs	SIR	95% CI
881	Packers	NIGW, PPWL	9,086	2	4.50	0.55, 16.30	8	1.14	0.49, 2.25
888	Furniture removers and porters	PPWL	312	0	0.00	0.00, 281.00	1	5.17	0.13, 28.80
889	Non-specified packing, freight handling, and storage work	PPWL	263	0	0.00	0.00, 233.00	1	3.81	0.10, 21.20
901	Firefighters	PPWL	8,126	0	0.00	0.00, 10.30	7	1.16	0.47, 2.39
903	Customs officials	NIGW, PPWL	4,024	0	0.00	0.00, 17.10	4	1.10	0.30, 2.81
904	Prison and reformatory officials	NIGW, PPWL	3,973	0	0.00	0.00, 18.70	4	1.21	0.33, 3.10
908	Civilian protective service work n.e.c.	NIGW, PPWL	12,117	0	0.00	0.00, 7.18	7	0.89	0.36, 1.83
909	Non-specified civilian protective service work	NIGW, PPWL	2	0	0.00	0.00, 18,129.00	0	0.00	0.00, 1,017.00
911	Catering supervisors	PPWL	7,067	0	0.00	0.00, 13.10	5	1.12	0.36, 2.60
912	Cooks	PPWL	5,888	0	0.00	0.00, 19.50	1	0.35	0.01, 1.97
913	Kitchen maids	PPWL	3,356	0	0.00	0.00, 38.60	2	1.49	0.18, 5.37
914	Nursemaids	NIGW, PPWL	1,427	0	0.00	0.00, 130.00	0	0.00	0.00, 10.90
915	Housekeeping service workers	PPWL, PPWL	1,306	0	0.00	0.00, 142.00	0	0.00	0.00, 13.10
916	Hotel receptionists	PPWL	1,018	0	0.00	0.00, 73.60	1	1.24	0.03, 6.89
917	Pursers, stewards, and stewardesses	IRAD, NIGW, PPWL	1,332	0	0.00	0.00, 61.40	1	1.03	0.03, 5.73
918	Housekeeping and related service work n.e.c.	PPWL	75	0	0.00	0.00, 1,914.00	0	0.00	0.00, 164.00
919	Non-specified housekeeping and related service work	PPWL	21	0	0.00	0.00, 5,590.00	0	0.00	0.00, 339.00
921	Waiters and waitresses	NIGW, PPWL	6,138	0	0.00	0.00, 15.60	3	0.80	0.17, 2.34
931	Building caretakers	PPWL	29,755	1	0.75	0.02, 4.18	25	1.22	0.79, 1.79
932	Cleaners	PPWL	11,668	0	0.00	0.00, 9.80	3	0.55	0.11, 1.62
939	Non-specified caretaking and cleaning work	PPWL	3	0	0.00	0.00, 26,846.00	0	0.00	0.00, 1,513.00
941	Hairdressers and beauticians	PPWL	7,056	0	0.00	0.00, 9.01	2	0.29	0.04, 1.06
942	Bath attendants	PPWL	625	0	0.00	0.00, 135.00	0	0.00	0.00, 8.34
944	Pressers	NOEXP	2,000	0	0.00	0.00, 29.80	2	0.95	0.12, 3.43
945	Coaches and horse trainers	PPWL, UV	2,275	0	0.00	0.00, 48.00	1	0.85	0.02, 4.72
946	Photographers	NIGW, UV	5,405	0	0.00	0.00, 14.00	3	0.68	0.14, 1.98
947	Undertakers	NIGW	587	0	0.00	0.00, 133.00	0	0.00	0.00, 8.50
948	Other service work n.e.c.	NIGW	2,047	0	0.00	0.00, 38.30	1	0.67	0.02, 3.72
949	Non-specified other service work	NIGW	3	0	0.00	0.00, 16,088.00	0	0.00	0.00, 937.00
981	Members of the armed forces	PPWL	25,245	2	1.48	0.18, 5.34	18	0.76	0.45, 1.21

SIR, standardized incidence ratio; NYK, Nordic Occupational Classification; N, number of persons in follow-up; ICD, International Classification of Diseases; Obs, observed; CI, confidence interval; n.e.c., not elsewhere classified.

[1]See Appendix 1 for exposure codes.
