# Supplementary material for: Occupation and mesothelioma in Sweden: updated incidence in men and women in the 27 years after the asbestos ban
Source: Epidemiol Health. 2016 Sep 20;38:e2016039. doi: 10.4178/epih.e2016039 (PMC5114438; doi:10.4178/epih.e2016039)
Supplement: Supplementary file 4 [file epih-38-e2016039-app4.pdf]

Appendix 4. Observed number of mesotheliomas in the peritoneum and pleura among women in Sweden in occupations exposed to chemical agents with SIRs from 1961 to 2009

NYK	Occupation title, 1980	Exposure code[1]	N	Peritoneum (ICD-7 158)			Pleura (ICD-7 162.2)		
				Obs	SIR	95% CI	Obs	SIR	95% CI
003	Mechanical engineers and technicians	DEEX	2,032	1	16.27	0.41, 90.70	0	0.00	0.00, 20.10
011	Chemists and physicists	BENZ, NIGW	3,438	0	0.00	0.00, 28.80	1	2.28	0.06, 12.70
014	Laboratory technicians and assistants	BENZ	11,149	2	7.28	0.88, 26.30	1	1.33	0.03, 7.44
021	Veterinarians	ANIM, PPWL, UV	443	0	0.00	0.00, 328.00	0	0.00	0.00, 126.00
045	Medical technicians	FORM, NIGW	5,133	1	4.42	0.11, 24.60	0	0.00	0.00, 4.90
338	Filling station attendants and demonstrators	BENZ, DEEX, GASO, NIGW, PPWL, TOLU	1,984	0	0.00	0.00, 38.60	0	0.00	0.00, 11.00
401	Working proprietors; agricultural, horticultural, and forestry enterprises	ANIM, ANIM, PPWL, UV	17,126	0	0.00	0.00, 3.89	3	1.25	0.26, 3.65
405	Livestock breeders	ANIM, FORM	367	0	0.00	0.00, 195.00	0	0.00	0.00, 74.50
406	Breeders of fur-bearing animals	ANIM, PPWL, UV	70	0	0.00	0.00, 1,053.00	0	0.00	0.00, 325.00
411	Agricultural and livestock workers	ANIM, ANIM, PPWL, UV	24,598	2	1.08	0.13, 3.90	1	0.17	0.00, 0.93
414	Fur-bearing animal farm workers	ANIM, PPWL, UV	80	0	0.00	0.00, 712.00	0	0.00	0.00, 214.00
418	Agricultural, horticultural and livestock work n.e.c.	ANIM, PPWL, UV	953	0	0.00	0.00, 130.00	0	0.00	0.00, 46.20
419	Non-specified agricultural, horticultural and livestock work	ANIM, PPWL, UV	2	0	0.00	0.00, 18,734.00	0	0.00	0.00, 10,479.00
441	Forest workers and log-drivers	FORM, PPWL, UV	939	0	0.00	0.00, 70.70	0	0.00	0.00, 22.10
501	Miners and quarrymen	BAP, DEEX, IRAD, PPWL, QUAR	65	0	0.00	0.00, 2,204.00	0	0.00	0.00, 753.00
502	Well drillers and diamond drillers	PPWL, QUAR, UV	5	0	0.00	0.00, 18,648.00	0	0.00	0.00, 6,483.00
503	Ore dressers	NI, PPWL, QUAR	126	0	0.00	0.00, 470.00	0	0.00	0.00, 139.00
504	Mining and quarrying work n.e.c.	DEEX, QUAR	66	0	0.00	0.00, 1,305.00	0	0.00	0.00, 358.00
509	Non-specified mining and quarrying work	DEEX, QUAR	2	0	0.00	0.00, 37,328.00	0	0.00	0.00, 7,804.00
631	Railway engine drivers and assistants	ASB, DEEX, NIGW	158	0	0.00	0.00, 1,106.00	0	0.00	0.00, 462.00
633	Motor-vehicle drivers and tram drivers	DEEX, NIGW	5,575	0	0.00	0.00, 19.20	1	1.63	0.04, 9.07
635	Deliverymen	DEEX, NIGW, PPWL, UV	3,810	0	0.00	0.00, 18.90	0	0.00	0.00, 5.92
636	Bus and tram conductors, traffic assistants	DEEX, NIGW, UV	892	0	0.00	0.00, 63.20	0	0.00	0.00, 17.50
639	Non-specified rail and road transport work	DEEX, NIGW, UV	6	0	0.00	0.00, 15,024.00	0	0.00	0.00, 4,794.00
641	Harbormasters	DEEX, NIGW	18	0	0.00	0.00, 8,744.00	0	0.00	0.00, 3,367.00
644	Road traffic supervisors	DEEX, NIGW	320	0	0.00	0.00, 436.00	0	0.00	0.00, 172.00
649	Non-specified traffic supervision	DEEX, NIGW	1	0	0.00	0.00, 145,405.00	0	0.00	0.00, 44,745.00
712	Fur tailors	ANIM	168	0	0.00	0.00, 307.00	0	0.00	0.00, 108.00
714	Upholsterers	BENZ, MCH, PPWL, TCE, TOLU, WOOD	748	0	0.00	0.00, 97.20	0	0.00	0.00, 30.20
715	Patternmakers and cutters	FORM	3,286	0	0.00	0.00, 14.50	1	1.21	0.03, 6.73
718	Sewing work n.e.c.	FORM	33,031	2	0.75	0.09, 2.70	18	2.14	1.27, 3.39
722	Shoe cutters, lasters, and sewers	BENZ, TOLU	3,662	0	0.00	0.00, 13.00	0	0.00	0.00, 4.21
729	Non-specified shoe and leather goods work	BENZ, TOLU	10	0	0.00	0.00, 5,613.00	0	0.00	0.00, 1,676.00
731	Furnacemen	ASB, BAP, CR, FE, NI, NIGW, PB, PPWL, QUAR, SO2, WELD	385	0	0.00	0.00, 361.00	0	0.00	0.00, 124.00
732	Metal annealers, temperers, and case-hardeners	BAP, CR, FE, NI, NIGW, PB, PPWL, WELD	68	0	0.00	0.00, 1,414.00	0	0.00	0.00, 436.00
733	Rolling mill workers	CR, CR, FE, NI, NI, NIGW, PPWL, SO2, WELD	214	0	0.00	0.00, 434.00	0	0.00	0.00, 121.00

(Continued to the next page)

Appendix 4. Continued

NYK	Occupation title, 1980	Exposure code[1]	N	Peritoneum (ICD-7 158)			Pleura (ICD-7 162.2)		
				Obs	SIR	95% CI	Obs	SIR	95% CI
735	Smiths and forgers	BAP, FE, NIGW, PB, PPWL, WELD	112	0	0.00	0.00, 1,117.00	0	0.00	0.00, 371.00
736	Metal casters and molders	BAP, CR, FE, FORM, NI, NIGW, PB, PPWL, QUAR, WELD	422	0	0.00	0.00, 227.00	0	0.00	0.00, 71.40
737	Wire and tube drawers	CR, FE, NI, NIGW, PPWL, WELD	200	0	0.00	0.00, 446.00	0	0.00	0.00, 131.00
738	Metal processing work n.e.c.	CR, FE, NI, PB, PPWL, QUAR, SO2, TCE, TRI, WELD	1,061	0	0.00	0.00, 63.10	0	0.00	0.00, 18.80
739	Non-specified metal processing work	CR, FE, NI, PB, PPWL, QUAR, SO2, TCE, TRI, WELD	133	0	0.00	0.00, 570.00	0	0.00	0.00, 158.00
750	Toolmakers, machine-tool setters, and operators	BAP, CR, FE, NI, PB, PPWL, TCE, TRI, WELD	10,824	1	2.47	0.06, 13.70	0	0.00	0.00, 2.85
751	Machinery fitters and machine assemblers	ASB, BAP, BENZ, CR, DEEX, FE, GASO, MCH, NI, PB, PPWL, TCE, TOLU, WELD	5,685	0	0.00	0.00, 25.10	1	2.38	0.06, 13.30
753	Sheet metal workers	ASB, CR, FE, NI, PB, PPWL, TRI, UV, WELD	381	0	0.00	0.00, 389.00	0	0.00	0.00, 142.00
754	Plumbers and pipe fitters	ASB, CR, FE, NI, PB, PPWL, WELD	76	0	0.00	0.00, 1,781.00	0	0.00	0.00, 617.00
755	Welders and flame cutters	ASB, BAP, CR, FE, FORM, NI, PB, PPWL, UV, WELD	1,464	0	0.00	0.00, 76.40	0	0.00	0.00, 24.40
757	Metal platers and coaters	CR, FE, FORM, NI, PB, PER, PPWL, TCE, TRI, WELD	351	0	0.00	0.00, 223.00	0	0.00	0.00, 72.10
759	Non-specified engineering and building metal work	CR, FE, NI, PB, PPWL, TCE, TRI, WELD	7,898	2	3.58	0.43, 12.90	1	0.53	0.01, 2.98
761	Electrical fitters and wiremen	ASB, PPWL	1,754	0	0.00	0.00, 86.80	0	0.00	0.00, 31.70
764	Radio and television assemblers and repairmen	MCH, PB, PER, TCE, TRI	4,994	0	0.00	0.00, 30.60	0	0.00	0.00, 11.40
766	Telephone and telegraph installers and repairmen	PB, PPWL, UV	1,247	0	0.00	0.00, 122.00	1	12.31	0.31, 68.60
769	Non-specified electrical and electronics work	ASB, FORM, MCH, PB, PER, TCE, TRI	6,396	0	0.00	0.00, 8.31	3	1.95	0.40, 5.71
771	Construction carpenters and joiners	ASB, PPWL, QUAR, UV, WOOD	122	0	0.00	0.00, 1,036.00	0	0.00	0.00, 333.00
772	Bench carpenters and cabinet makers	FORM, PPWL, WOOD	2,884	0	0.00	0.00, 34.60	0	0.00	0.00, 11.00
773	Laminated wood and fiberboard workers	CR, FORM, PPWL, WOOD	219	0	0.00	0.00, 808.00	0	0.00	0.00, 341.00
774	Frame and circular sawyers and planers	CR, NIGW, PPWL, WOOD	333	0	0.00	0.00, 357.00	0	0.00	0.00, 118.00
778	Wood work n.e.c.	PPWL, WOOD	889	0	0.00	0.00, 70.90	0	0.00	0.00, 21.30
779	Non-specified wood work	PPWL, WOOD	272	0	0.00	0.00, 204.00	0	0.00	0.00, 61.30
781	Painters	ASB, BENZ, CR, FORM, MCH, PB, PPWL, TCE, TOLU, UV	1,173	0	0.00	0.00, 70.60	0	0.00	0.00, 22.90
791	Bricklayers	ASB, PPWL, QUAR, UV	12	0	0.00	0.00, 13,562.00	0	0.00	0.00, 5,361.00
793	Concrete and construction workers	ASB, PPWL, QUAR, UV	121	0	0.00	0.00, 1,007.00	0	0.00	0.00, 361.00
794	Insulators	ASB, BAP, BITU, PPWL, UV	23	0	0.00	0.00, 5,568.00	0	0.00	0.00, 1,619.00
801	Typographers and lithographers	PB	4,036	0	0.00	0.00, 26.10	0	0.00	0.00, 8.92
806	Bookbinders	MCH, PPWL	4,544	1	3.38	0.09, 18.90	2	2.17	0.26, 7.84
808	Printing work n.e.c.	BENZ, GASO, MCH, PB, PPWL, TCE, TOLU	872	0	0.00	0.00, 87.00	1	7.15	0.18, 39.80
809	Non-specified printing work	BENZ, GASO, PB, TOLU, TRI	30	0	0.00	0.00, 1,643.00	0	0.00	0.00, 516.00
811	Glass formers and cutters	ASB, NI, NIGW, PB, PPWL, QUAR	312	0	0.00	0.00, 188.00	0	0.00	0.00, 56.60
812	Potters	NI, NIGW, PB, PPWL, QUAR	524	0	0.00	0.00, 121.00	1	10.10	0.26, 56.30
813	Glass and ceramics kilnmen	NI, NIGW, PB, PPWL, QUAR, SO2	45	0	0.00	0.00, 1,044.00	0	0.00	0.00, 341.00
814	Glass, china, and ceramics painters and decorators	NI, NIGW, PB, QUAR	465	0	0.00	0.00, 120.00	0	0.00	0.00, 38.80

(Continued to the next page)

Appendix 4. Continued

NYK	Occupation title, 1980	Exposure code[1]	N	Peritoneum (ICD-7 158)			Pleura (ICD-7 162.2)		
				Obs	SIR	95% CI	Obs	SIR	95% CI
818	Glass, pottery, and tile work n.e.c.	NIGW, QUAR	889	0	0.00	0.00, 71.10	0	0.00	0.00, 23.70
819	Non-specified glass, pottery, and tile work	NIGW, QUAR	699	0	0.00	0.00, 79.40	0	0.00	0.00, 24.50
831	Chemical process workers	BENZ, MCH, NIGW, PER, PPWL, TOLU, TRI	753	0	0.00	0.00, 107.00	0	0.00	0.00, 32.50
834	Paper pulp workers	NIGW, SO2	258	0	0.00	0.00, 376.00	0	0.00	0.00, 117.00
836	Paper and paperboard workers	ASB, FORM, NIGW, PPWL	3,266	0	0.00	0.00, 18.20	1	1.58	0.04, 8.78
838	Chemical and cellulose processing work n.e.c.	ASB, BENZ, BITU, FORM, MCH, NIGW, PB, PPWL, QUAR, TOLU	680	0	0.00	0.00, 116.00	0	0.00	0.00, 38.40
839	Non-specified chemical and cellulose processing work	NIGW, SO2	1,532	1	11.48	0.29, 64.00	0	0.00	0.00, 12.90
851	Rubber products workers	BENZ, NIGW, PPWL, TCE, TOLU, TRI	2,596	0	0.00	0.00, 25.90	0	0.00	0.00, 7.96
852	Plastic products workers	FORM, MCH, NIGW, PB, PPWL, TCE	4,531	1	4.88	0.12, 27.20	2	2.94	0.36, 10.60
853	Tanners and fur dressers	CR, NIGW, PPWL	172	0	0.00	0.00, 341.00	0	0.00	0.00, 112.00
856	Stone cutters and carvers	NIGW, PPWL, QUAR	19	0	0.00	0.00, 6,740.00	0	0.00	0.00, 2,075.00
857	Paper and paperboard products workers	FORM, NIGW, PPWL	2,872	0	0.00	0.00, 20.80	1	1.77	0.04, 9.86
871	Stationary engine and related equipment operators	DEEX, NIGW, SO2	33	0	0.00	0.00, 4,541.00	0	0.00	0.00, 1,525.00
872	Crane and hoist operators	ASB, NIGW	1,483	0	0.00	0.00, 57.40	1	4.40	0.11, 24.50
873	Riggers and cable splicers	ASB, NI, PPWL	2	0	0.00	0.00, 55,230.00	0	0.00	0.00, 17,711.00
874	Construction machine operators	BITU, DEEX, PPWL	55	0	0.00	0.00, 2,367.00	0	0.00	0.00, 778.00
875	Truck and conveyor operators	DEEX, NIGW, PPWL	1,105	0	0.00	0.00, 114.00	0	0.00	0.00, 36.90
876	Greasers	DEEX, PPWL, TOLU	93	0	0.00	0.00, 1,469.00	0	0.00	0.00, 508.00
882	Dockers and freight handlers	DEEX, PPWL, UV	84	0	0.00	0.00, 1,229.00	0	0.00	0.00, 413.00
883	Store and warehouse workers	ASB, PPWL	10,051	1	1.75	0.04, 9.77	1	0.57	0.01, 3.15
902	Policemen	DEEX, NIGW, PB, PPWL	1,631	0	0.00	0.00, 96.30	0	0.00	0.00, 36.40
933	Chimney sweeps	ASB, BAP, PPWL	7	0	0.00	0.00, 20,338.00	0	0.00	0.00, 6,611.00
943	Launderers and dry-cleaners	BENZ, PER, PPWL, TCE, TRI	10,307	0	0.00	0.00, 4.87	0	0.00	0.00, 1.64

SIR, standardized incidence ratio; NYK, Nordic Occupational Classification; N, number of persons in follow-up; ICD, International Classification of Diseases; Obs, observed; CI, confidence interval; n.e.c., not elsewhere classified.

[1]See Appendix 1 for exposure codes.
