# Supplementary material for: Occupation and mesothelioma in Sweden: updated incidence in men and women in the 27 years after the asbestos ban
Source: Epidemiol Health. 2016 Sep 20;38:e2016039. doi: 10.4178/epih.e2016039 (PMC5114438; doi:10.4178/epih.e2016039)
Supplement: Supplementary file 5 [file epih-38-e2016039-app5.pdf]

Appendix 5. Observed number of mesotheliomas in the peritoneum and pleura among women in Sweden in occupations not exposed to chemical agents with SIRs from 1961 to 2009

NYK	Occupation title, 1980	Exposure code[1]	N	Peritoneum (ICD-7 158)			Pleura (ICD-7 162.2)		
				Obs	SIR	95% CI	Obs	SIR	95% CI
001	Architects, building and construction engineers and technicians	NOEXP	3,025	0	0.00	0.00, 39.90	0	0.00	0.00, 12.80
002	Electrical, electronics, and telecommunications engineers and technicians	NOEXP	2,207	0	0.00	0.00, 61.40	0	0.00	0.00, 22.20
004	Chemical engineers and technicians	NOEXP	1,649	0	0.00	0.00, 70.30	0	0.00	0.00, 24.40
005	Metallurgists and mining engineers and technicians	NOEXP	141	0	0.00	0.00, 969.00	0	0.00	0.00, 336.00
006	Engineers and technicians n.e.c.	NOEXP	1,805	0	0.00	0.00, 43.80	0	0.00	0.00, 14.70
007	Surveyors, measurers, and cartographers	UV	268	0	0.00	0.00, 548.00	0	0.00	0.00, 206.00
008	Technical assistants	NOEXP	8,140	0	0.00	0.00, 8.29	1	0.67	0.02, 3.76
009	Non-specified engineering work	NOEXP	638	0	0.00	0.00, 271.00	0	0.00	0.00, 118.00
013	Geologists and meteorologists	UV	288	0	0.00	0.00, 328.00	0	0.00	0.00, 97.90
019	Non-specified chemical and physical work	NOEXP	49	0	0.00	0.00, 1,131.00	0	0.00	0.00, 285.00
022	Biologists	UV	617	0	0.00	0.00, 228.00	0	0.00	0.00, 89.30
023	Agricultural and horticultural researchers/advisors	UV	940	0	0.00	0.00, 114.00	0	0.00	0.00, 41.30
024	Forestry researchers/advisors	PPWL, UV	49	0	0.00	0.00, 3,416.00	0	0.00	0.00, 1,394.00
031	Physicians and surgeons	NOEXP	7,265	0	0.00	0.00, 15.50	2	2.76	0.33, 9.96
032	Dentists	NOEXP	3,984	1	5.89	0.15, 32.80	0	0.00	0.00, 7.02
040	Registered nurses	NIGW, PPWL	61,685	3	1.12	0.23, 3.28	8	0.95	0.41, 1.87
041	Midwives	NIGW, PPWL	4,565	0	0.00	0.00, 19.40	0	0.00	0.00, 6.71
042	Attendants in psychiatric care	NIGW	13,596	0	0.00	0.00, 6.18	0	0.00	0.00, 1.91
043	Practical nurses and hospital orderlies	NIGW, PPWL	125,497	5	1.02	0.33, 2.38	16	1.04	0.60, 1.69
044	Dental nurses	NOEXP	14,563	0	0.00	0.00, 7.47	2	1.31	0.16, 4.75
046	Pharmacists	NIGW	4,083	0	0.00	0.00, 19.00	0	0.00	0.00, 5.77
047	Physiotherapists and occupational therapists	PPWL	13,419	0	0.00	0.00, 8.27	3	2.32	0.48, 6.77
048	Health and nursing work n.e.c.	NIGW, PPWL	1,085	0	0.00	0.00, 134.00	0	0.00	0.00, 52.50
049	Non-specified health and nursing work	NIGW, PPWL	31	0	0.00	0.00, 2,249.00	0	0.00	0.00, 750.00
050	Principals and headmasters	NOEXP	905	0	0.00	0.00, 75.50	0	0.00	0.00, 26.00
051	University and higher education teachers	NOEXP	3,650	0	0.00	0.00, 32.50	0	0.00	0.00, 10.20
052	Teachers of theoretical subjects	PPWL	27,148	3	2.56	0.53, 7.48	1	0.26	0.01, 1.45
053	Schoolmasters	PPWL	56,890	5	1.59	0.52, 3.71	11	1.20	0.60, 2.14
054	Teachers of painting, music, and physical education	PPWL	17,479	2	2.13	0.26, 7.68	5	1.73	0.56, 4.04
055	Teachers of vocational subjects	PPWL	3,938	1	4.53	0.11, 25.20	0	0.00	0.00, 4.87
056	Pre-primary education teachers	PPWL, UV	33,027	2	2.32	0.28, 8.37	2	0.85	0.10, 3.06
057	Educational methods advisors	NOEXP	2,739	0	0.00	0.00, 35.30	0	0.00	0.00, 11.20
058	Educational work n.e.c.	NOEXP	5,177	0	0.00	0.00, 25.00	0	0.00	0.00, 8.52
059	Non-specified educational work	NOEXP	443	0	0.00	0.00, 235.00	0	0.00	0.00, 79.10
061	Ministers and priests	NOEXP	1,880	0	0.00	0.00, 32.90	0	0.00	0.00, 10.50
068	Religious work n.e.c.	NOEXP	2,097	0	0.00	0.00, 45.00	0	0.00	0.00, 15.20
069	Non-specified religious work	NOEXP	5	0	0.00	0.00, 12,218.00	0	0.00	0.00, 3,253.00
071	Judges and other lawyers in courts of law	NOEXP	676	0	0.00	0.00, 183.00	0	0.00	0.00, 60.00

(Continued to the next page)

Appendix 5. Continued

NYK	Occupation title, 1980	Exposure code[1]	N	Peritoneum (ICD-7 158)			Pleura (ICD-7 162.2)		
				Obs	SIR	95% CI	Obs	SIR	95% CI
072	Prosecutors and senior police officers	NOEXP	311	0	0.00	0.00, 444.00	0	0.00	0.00, 163.00
073	Lawyers in private practice	NOEXP	404	0	0.00	0.00, 258.00	0	0.00	0.00, 83.80
074	Corporation and organization lawyers	NOEXP	748	0	0.00	0.00, 208.00	0	0.00	0.00, 81.70
078	Legal work n.e.c.	NOEXP	36	0	0.00	0.00, 2,687.00	0	0.00	0.00, 752.00
079	Non-specified legal work	NOEXP	8	0	0.00	0.00, 5,157.00	0	0.00	0.00, 1,603.00
081	Sculptors, painters, photographers, and commercial artists	NOEXP	3,164	0	0.00	0.00, 30.20	0	0.00	0.00, 9.98
082	Designers	NOEXP	2,175	0	0.00	0.00, 44.30	0	0.00	0.00, 14.00
083	Display artists	NOEXP	912	0	0.00	0.00, 112.00	0	0.00	0.00, 36.10
084	Authors	NOEXP	253	0	0.00	0.00, 251.00	0	0.00	0.00, 89.50
085	Journalists and editors	NOEXP	5,321	1	5.04	0.13, 28.10	0	0.00	0.00, 6.00
086	Performing artists	NOEXP	2,052	0	0.00	0.00, 45.80	1	3.95	0.10, 22.00
087	Composers and musicians	NOEXP	1,299	0	0.00	0.00, 75.40	1	7.15	0.18, 39.80
088	Literary and artistic work n.e.c.	NOEXP	1,157	0	0.00	0.00, 94.00	1	7.89	0.20, 44.00
089	Non-specified literary and artistic work	NOEXP	28	0	0.00	0.00, 2,183.00	0	0.00	0.00, 697.00
091	Accountants and auditors	NIGW	2,415	0	0.00	0.00, 52.80	0	0.00	0.00, 19.20
092	Social workers	NIGW	28,010	1	1.06	0.03, 5.92	3	1.09	0.23, 3.20
093	Librarians, archivists, and curators	NOEXP	7,906	0	0.00	0.00, 10.50	2	1.83	0.22, 6.61
094	Economists and statisticians	NOEXP	5,125	0	0.00	0.00, 34.60	0	0.00	0.00, 15.20
095	Psychologists	NIGW	3,882	0	0.00	0.00, 21.30	1	1.78	0.05, 9.94
096	Staff officers	NOEXP	9,685	0	0.00	0.00, 14.60	1	1.40	0.04, 7.78
097	Systems analysts and programmers	NIGW	4,806	0	0.00	0.00, 35.40	0	0.00	0.00, 14.10
098	Other related work	NOEXP	2,389	1	11.81	0.30, 65.80	0	0.00	0.00, 14.20
099	Non-specified other professional, technical, and related work	NOEXP	16	0	0.00	0.00, 6,989.00	0	0.00	0.00, 2,067.00
101	Government legislative and administrative work	NOEXP	10,921	0	0.00	0.00, 10.00	0	0.00	0.00, 3.35
111	General managers	NOEXP	2,404	0	0.00	0.00, 23.20	0	0.00	0.00, 8.73
118	Other business managers, including managers with specific functions	NOEXP	11,359	0	0.00	0.00, 8.10	0	0.00	0.00, 2.74
119	Non-specified business administrative and other technical and economical administrative work	NOEXP	1	0	0.00	0.00, 80,140.00	0	0.00	0.00, 14,685.00
201	Bookkeepers and office cashiers	NOEXP	55,066	3	1.00	0.21, 2.93	9	0.92	0.42, 1.75
203	Bank tellers	NOEXP	3,891	0	0.00	0.00, 17.70	0	0.00	0.00, 5.16
204	Cashiers in retail stores and restaurants	PPWL	17,049	0	0.00	0.00, 4.17	1	0.35	0.01, 1.95
208	Debt collectors	NOEXP	311	0	0.00	0.00, 344.00	0	0.00	0.00, 111.00
209	Non-specified bookkeeping and clerical work	NOEXP	44	0	0.00	0.00, 872.00	0	0.00	0.00, 333.00
290	Secretaries, typists, and related workers	NOEXP	156,010	6	1.07	0.39, 2.33	23	1.30	0.82, 1.95
291	Computer operators	NIGW	10,485	1	2.42	0.06, 13.50	2	1.43	0.17, 5.16
292	Bank employees (general bank work)	NOEXP	13,696	2	4.04	0.49, 14.60	0	0.00	0.00, 2.39
293	Travel agency employees	NOEXP	2,524	0	0.00	0.00, 49.50	1	4.57	0.12, 25.50
294	Forwarding and shipping agents	NOEXP	1,296	0	0.00	0.00, 85.20	0	0.00	0.00, 30.90
295	Property managers and store managers	NOEXP	1,879	0	0.00	0.00, 38.40	0	0.00	0.00, 13.70

(Continued to the next page)

Appendix 5. Continued

NYK	Occupation title, 1980	Exposure code[1]	N	Peritoneum (ICD-7 158)			Pleura (ICD-7 162.2)		
				Obs	SIR	95% CI	Obs	SIR	95% CI
296	Insurance raters and claims adjusters	NOEXP	5,757	0	0.00	0.00, 12.30	1	1.04	0.03, 5.78
297	Employees in national insurance offices	NOEXP	8,271	1	3.65	0.09, 20.40	2	2.39	0.29, 8.63
298	Cost accountants and estimating clerks	NOEXP	18,173	2	1.27	0.15, 4.57	9	1.79	0.82, 3.40
299	Non-specified clerical work	NOEXP	36,587	5	1.98	0.64, 4.63	5	0.56	0.18, 1.30
301	Working proprietors, wholesale trade	NOEXP	818	0	0.00	0.00, 94.80	0	0.00	0.00, 36.00
302	Working proprietors, retail trade	NOEXP	19,665	1	0.72	0.02, 4.00	2	0.53	0.06, 1.91
309	Non-specified working proprietors	NOEXP	71	0	0.00	0.00, 2,296.00	0	0.00	0.00, 1,210.00
311	Insurance representatives and agents	NOEXP	468	0	0.00	0.00, 190.00	0	0.00	0.00, 67.30
312	Brokers and valuers	NOEXP	759	0	0.00	0.00, 159.00	0	0.00	0.00, 61.50
313	Advertising salesmen	NOEXP	3,442	0	0.00	0.00, 40.70	1	3.98	0.10, 22.20
331	Commercial travelers, buyers, and dealers	NOEXP	16,425	0	0.00	0.00, 6.20	2	1.12	0.14, 4.04
332	Shop managers	PPWL	10,619	0	0.00	0.00, 4.83	5	2.19	0.71, 5.12
333	Shop assistants	PPWL	127,312	12	1.49	0.77, 2.59	30	1.13	0.76, 1.61
339	Non-specified other sales work	NOEXP	14	0	0.00	0.00, 5,845.00	0	0.00	0.00, 1,695.00
402	Farm managers and supervisors	PPWL, UV	89	0	0.00	0.00, 1,237.00	0	0.00	0.00, 458.00
403	Forestry managers and supervisors	UV	104	0	0.00	0.00, 1,129.00	0	0.00	0.00, 358.00
404	Horticultural managers and supervisors	NIGW, UV	190	0	0.00	0.00, 574.00	0	0.00	0.00, 221.00
407	Reindeer owners	PPWL, UV	35	0	0.00	0.00, 2,135.00	0	0.00	0.00, 921.00
409	Non-specified agricultural, horticultural, and forestry management work	PPWL, UV	1	0	0.00	0.00, 38,198.00	0	0.00	0.00, 10,007.00
412	Horticultural workers	PPWL, UV	6,005	0	0.00	0.00, 8.90	1	0.79	0.02, 4.40
415	Reindeer herdsmen	UV	29	0	0.00	0.00, 1,762.00	0	0.00	0.00, 648.00
421	Game-keepers and hunters	UV	7	0	0.00	0.00, 23,437.00	0	0.00	0.00, 7,829.00
431	Fishermen	PPWL, UV	105	0	0.00	0.00, 613.00	0	0.00	0.00, 208.00
432	Fish-breeders	PPWL, UV	37	0	0.00	0.00, 1,925.00	0	0.00	0.00, 649.00
601	Ships' deck officers	NIGW, UV	9	0	0.00	0.00, 8,850.00	0	0.00	0.00, 3,018.00
602	Ship pilots	NIGW, UV	1	0	0.00	0.00, 109,892.00	0	0.00	0.00, 32,275.00
603	Ships' engineers	NIGW, UV	5	0	0.00	0.00, 35,765.00	0	0.00	0.00, 15,712.00
609	Non-specified ships' officers	NIGW, UV	1	0	0.00	0.00, 349,361.00	0	0.00	0.00, 162,002.00
611	Ships' deck and engine-room crew	NIGW, PPWL, UV	76	0	0.00	0.00, 2,508.00	0	0.00	0.00, 1,012.00
621	Aircraft pilots, navigators, and flight engineers	IRAD, NIGW	20	0	0.00	0.00, 8,083.00	0	0.00	0.00, 2,823.00
632	Railway guards	NIGW, UV	592	0	0.00	0.00, 277.00	0	0.00	0.00, 110.00
642	Air traffic controllers and flight dispatchers	NIGW	1,186	0	0.00	0.00, 140.00	0	0.00	0.00, 54.80
643	Railway station masters and train dispatchers	NIGW	452	0	0.00	0.00, 170.00	0	0.00	0.00, 56.90
651	Post-office clerks	NOEXP	13,510	1	1.39	0.04, 7.77	2	0.88	0.11, 3.16
652	Telecommunications traffic officers	NOEXP	192	0	0.00	0.00, 351.00	0	0.00	0.00, 120.00
653	Telephone operators	NOEXP	23,067	3	1.96	0.40, 5.74	7	1.40	0.56, 2.88
655	Telegraph and radio operators	NOEXP	1,994	0	0.00	0.00, 31.90	1	2.90	0.07, 16.10
659	Non-specified post and telecommunications work	NOEXP	6	0	0.00	0.00, 5,772.00	0	0.00	0.00, 2,440.00
661	Sorting clerks and postal workers	NIGW, PPWL, UV	6,028	2	9.27	1.12, 33.50	1	1.47	0.04, 8.21
662	Messengers	NIGW, PPWL, UV	1,300	0	0.00	0.00, 71.90	0	0.00	0.00, 24.30

(Continued to the next page)

Appendix 5. Continued

NYK	Occupation title, 1980	Exposure code[1]	N	Peritoneum (ICD-7 158)			Pleura (ICD-7 162.2)		
				Obs	SIR	95% CI	Obs	SIR	95% CI
671	Lighthouse and lock operators, ferry and harbor service assistants	NIGW, UV	53	0	0.00	0.00, 1,484.00	0	0.00	0.00, 488.00
678	Railway linemen	NIGW, UV	248	0	0.00	0.00, 205.00	0	0.00	0.00, 68.20
699	Non-specified transport and communications work	NIGW, UV	5	0	0.00	0.00, 13,319.00	0	0.00	0.00, 3,922.00
701	Spinners, weavers, knitters, and dyers	NIGW	15,722	2	1.66	0.20, 5.99	4	1.11	0.30, 2.84
711	Tailors and dressmakers	NOEXP	11,440	0	0.00	0.00, 4.98	4	2.00	0.55, 5.12
713	Milliners and hat makers	NOEXP	2,263	0	0.00	0.00, 18.90	0	0.00	0.00, 6.43
719	Non-specified sewing work	NIGW	462	0	0.00	0.00, 124.00	0	0.00	0.00, 39.00
721	Shoemakers and shoe repairers	NOEXP	100	0	0.00	0.00, 739.00	0	0.00	0.00, 250.00
726	Leather goods makers	NOEXP	1,226	0	0.00	0.00, 43.80	0	0.00	0.00, 12.80
741	Precision-tool makers	NIGW	1,020	0	0.00	0.00, 102.00	1	8.84	0.22, 49.30
742	Watchmakers	NOEXP	53	0	0.00	0.00, 1,666.00	0	0.00	0.00, 518.00
743	Opticians	NOEXP	591	0	0.00	0.00, 229.00	0	0.00	0.00, 85.90
744	Dental technicians	NOEXP	1,574	0	0.00	0.00, 53.90	0	0.00	0.00, 17.10
745	Goldsmiths and silversmiths	NOEXP	390	0	0.00	0.00, 172.00	0	0.00	0.00, 58.50
749	Non-specified precision-tool manufacturing work	NOEXP	10	0	0.00	0.00, 5,704.00	0	0.00	0.00, 1,617.00
765	Recording, sound, and light equipment operators	NOEXP	147	0	0.00	0.00, 1,187.00	0	0.00	0.00, 501.00
795	Glaziers	PPWL, UV	99	0	0.00	0.00, 1,149.00	0	0.00	0.00, 401.00
797	Divers and pipe layers	PPWL	7	0	0.00	0.00, 20,442.00	0	0.00	0.00, 7,486.00
821	Grain mill and oil press workers	PPWL	62	0	0.00	0.00, 1,237.00	0	0.00	0.00, 400.00
822	Bakers and pastry cooks	PPWL	6,126	0	0.00	0.00, 8.11	2	1.49	0.18, 5.39
823	Chocolate and confectionary workers	PPWL	1,823	0	0.00	0.00, 32.10	0	0.00	0.00, 10.60
824	Brewery, distillery, and other beverage plant workers	PPWL	979	0	0.00	0.00, 51.70	1	5.06	0.13, 28.20
825	Canning workers	PPWL	3,882	0	0.00	0.00, 13.40	4	4.90	1.33, 12.50
826	Butchers and meat preparers	PPWL	1,989	0	0.00	0.00, 34.00	1	3.01	0.08, 16.80
827	Dairy workers	PPWL	918	0	0.00	0.00, 56.70	0	0.00	0.00, 18.90
828	Food processing work n.e.c.	PPWL	1,492	0	0.00	0.00, 48.20	0	0.00	0.00, 15.60
829	Non-specified food processing work	PPWL	21	0	0.00	0.00, 2,444.00	0	0.00	0.00, 818.00
841	Tobacco workers	NIGW, PPWL	680	0	0.00	0.00, 85.50	0	0.00	0.00, 25.90
850	Basketry weavers	NIGW	47	0	0.00	0.00, 951.00	0	0.00	0.00, 351.00
854	Photographic laboratory workers	PPWL	1,105	0	0.00	0.00, 67.40	0	0.00	0.00, 20.80
855	Musical instrument makers and tuners	NIGW	44	0	0.00	0.00, 1,444.00	0	0.00	0.00, 498.00
858	Other production and related work n.e.c.	PPWL	3,641	0	0.00	0.00, 17.60	1	1.55	0.04, 8.62
859	Non-specified other production and related work	PPWL	1	0	0.00	0.00, 154,749.00	0	0.00	0.00, 49,892.00
861	Unskilled manual workers	PPWL	3,258	0	0.00	0.00, 15.30	0	0.00	0.00, 5.62
881	Packers	NIGW, PPWL	15,318	4	4.49	1.22, 11.50	3	1.07	0.22, 3.13
888	Furniture removers and porters	PPWL	1	0	0.00	0.00, 9,705,438.00	0	0.00	0.00, 9,705,438.00
889	Non-specified packing, freight handling, and storage work	PPWL	111	0	0.00	0.00, 445.00	0	0.00	0.00, 138.00
901	Firefighters	PPWL	15	0	0.00	0.00, 8,983.00	0	0.00	0.00, 2,954.00
903	Customs officials	NIGW, PPWL	298	0	0.00	0.00, 595.00	0	0.00	0.00, 256.00
904	Prison and reformatory officials	NIGW, PPWL	1,393	0	0.00	0.00, 105.00	0	0.00	0.00, 44.70

(Continued to the next page)

Appendix 5. Continued

NYK	Occupation title, 1980	Exposure code[1]	N	Peritoneum (ICD-7 158)			Pleura (ICD-7 162.2)		
				Obs	SIR	95% CI	Obs	SIR	95% CI
908	Civilian protective service work n.e.c.	NIGW, PPWL	1,946	0	0.00	0.00, 50.10	1	4.54	0.12, 25.30
911	Catering supervisors	PPWL	14,586	0	0.00	0.00, 3.43	4	1.32	0.36, 3.38
912	Cooks	PPWL	18,248	1	0.88	0.02, 4.90	2	0.65	0.08, 2.33
913	Kitchen maids	PPWL	34,249	3	1.64	0.34, 4.80	4	0.72	0.20, 1.84
914	Nursemaids	NIGW, PPWL	72,368	1	0.41	0.01, 2.27	6	0.84	0.31, 1.82
915	Housekeeping service workers	PPWL, PPWL	67,042	2	0.52	0.06, 1.87	4	0.40	0.11, 1.02
916	Hotel receptionists	PPWL	1,097	0	0.00	0.00,100.00	1	9.07	0.23, 50.50
917	Pursers, stewards, and stewardesses	IRAD, NIGW, PPWL	3,237	0	0.00	0.00, 34.50	0	0.00	0.00, 11.90
918	Housekeeping and related service work n.e.c.	PPWL	1,770	0	0.00	0.00, 31.00	0	0.00	0.00, 11.80
919	Non-specified housekeeping and related service work	PPWL	57	0	0.00	0.00, 965.00	0	0.00	0.00, 352.00
921	Waiters and waitresses	NIGW, PPWL	31,531	4	1.88	0.51, 4.82	8	1.19	0.51, 2.34
931	Building caretakers	PPWL	3,728	0	0.00	0.00, 15.00	1	1.50	0.04, 8.34
932	Cleaners	PPWL	103,538	7	1.21	0.49, 2.49	27	1.59	1.05, 2.31
939	Non-specified caretaking and cleaning work	PPWL	4	0	0.00	0.00, 8,805.00	0	0.00	0.00, 3,307.00
941	Hairdressers and beauticians	PPWL	18,912	1	1.06	0.03, 5.92	2	0.64	0.08, 2.31
942	Bath attendants	PPWL	1,625	0	0.00	0.00, 31.80	1	3.05	0.08, 17.00
944	Pressers	NOEXP	4,387	0	0.00	0.00, 9.92	3	2.76	0.57, 8.08
945	Coaches and horse trainers	PPWL, UV	814	0	0.00	0.00, 157.00	0	0.00	0.00, 53.60
946	Photographers	NIGW, UV	1,098	0	0.00	0.00, 58.80	1	5.09	0.13, 28.40
947	Undertakers	NIGW	159	0	0.00	0.00, 376.00	0	0.00	0.00, 145.00
948	Other service work n.e.c.	NIGW	2,141	0	0.00	0.00, 35.60	0	0.00	0.00, 11.80
949	Non-specified other service work	NIGW	4	0	0.00	0.00, 21,285.00	0	0.00	0.00, 5,925.00
981	Members of the armed forces	PPWL	204	0	0.00	0.00, 712.00	0	0.00	0.00, 251.00

SIR, standardized incidence ratio; NYK, Nordic Occupational Classification; N, number of persons in follow-up; ICD, International Classification of Diseases; Obs, observed; CI, confidence interval; n.e.c., not elsewhere classified.

[1]See Appendix 1 for exposure codes.
